# Cancer risk in heart or lung transplant recipients: A comprehensive analysis of 21 prospective cohorts

**DOI:** 10.1002/cam4.3525

**Published:** 2020-10-13

**Authors:** Fan Ge, Caichen Li, Xin Xu, Zhenyu Huo, Runchen Wang, Yaokai Wen, Haoxin Peng, Xiangrong Wu, Hengrui Liang, Guilin Peng, Run Li, Danxia Huang, Ying Chen, Shan Xiong, Ran Zhong, Bo Cheng, Jianfu Li, Jianxing He, Wenhua Liang

**Affiliations:** ^1^ Department of Thoracic Surgery and Oncology China State Key Laboratory of Respiratory Disease & National Clinical Research Center for Respiratory Disease the First Affiliated Hospital of Guangzhou Medical University Guangzhou China; ^2^ First Clinical School Guangzhou Medical University Guangzhou China; ^3^ Department of Transplantation the First Affiliated Hospital of Guangzhou Medical University Guangzhou China; ^4^ Nanshan School Guangzhou Medical University Guangzhou China

**Keywords:** cancer risk, heart transplantation, lung transplantation, meta‐analysis, tumor mutation burden

## Abstract

We performed a meta‐analysis to determine cancer risks at multiple sites and their associations with tumor mutation burden (TMB), an index for immunogenicity, in heart or lung transplant recipients. A comprehensive search of PubMed, Web of Science, EMBASE, and Medline was conducted. Random effects models were used to calculate standardized incidence ratios (SIRs) versus the general population and to determine the risks of different cancers. Weighted linear regression (WLR) was used to analyze the associations between the SIRs and TMBs. (PROSPERO CRD42020159599). Data from 21 studies including 116,438 transplant recipients (51,173 heart transplant recipients and 65,265 lung transplant recipients) with a total follow‐up of 601,330.7 person‐years were analyzed. Compared with the general population, heart transplant recipients displayed a 3.13‐fold higher cancer risk [SIR: 3.13; 95% confidence interval (CI): 2.38–4.13; *p* < 0.001]; lung transplant recipients displayed a 4.28‐fold higher cancer risk [SIR: 4.28; 95% CI: 3.18–5.77; *p* < 0.001]. The correlation coefficients were 0.54 (*p* = 0.049) and 0.79 (*p* < 0.001) in heart and lung transplant recipients, respectively, indicating that 29% and 63% of the differences in the SIRs for cancer types might be explained by the TMBs. Our study demonstrated that both heart and lung transplant recipients displayed a higher risk of certain site‐specific cancers. These findings can provide individualized guidance for clinicians for detection of cancer among heart or lung transplantation recipients. In addition, we provided evidence that increased risks of post‐transplant cancers can be attributed to immunosuppression.

## Graphical abstract

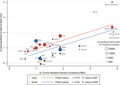

## 1 INTRODUCTION

Heart or lung transplantation is recognized as the best treatment option to improve the quality of life and survival of patients with some end‐stage cardiac or pulmonary diseases, such as heart failure, chronic obstructive pulmonary disease, and idiopathic pulmonary fibrosis. According to the International Society for Heart and Lung Transplantation (ISHLT),^1^ heart transplant recipients have a median survival of more than 12 years and lung transplant recipients who survived past the first year after primary transplant, have a median survival of 8.7 years.

Nevertheless, compared with the general population, heart or lung transplant recipients are at higher risk for cancers. In accordance with the ISHLT, 16% of all 5‐year‐survivors and 28% of 10‐year‐survivors were diagnosed with at least one post‐heart transplantation cancer and 17.3% of the post‐lung transplantation recipients died from malignancies 5–10 years after transplantation.^1^, ^2^, ^3^ Compared with the general population, the cancer risk of liver or kidney transplant recipients has increased by 2 to 4 times, and the cancer risk of thoracic organs is even higher.^4^, ^5^, ^6^, ^7^, ^8^ Though exerting great efficacy in the extension of survival in solid organ transplantations, immunosuppression is considered to be an important inducement of tumors after organ transplantation.^9^, ^10^, ^11^, ^12^, ^13^, ^14^, ^15^

Tumor mutation burden (TMB) is defined as the total number of somatic gene coding errors, base substitution, gene insertion or deletion errors detected in every million bases.^16^ The diversity of TMB and cancer types reflect the different immunogenicity, which is intimately related to the ability of the immune system to recognize tumors. As a result, this may be related to the risks of multiple sites of heart or lung transplant recipients.

Herein, we performed a large‐scale examination of prospective cohort studies and conducted a meta‐analysis to determine the risks of overall cancer, and each site‐specific cancer of heart or lung transplant recipients and determine which cancer type has the highest risk. We also compared these associations among recipients with different baseline characteristics and used weighted linear regression (WLR) to analyze the associations between corresponding standardized incidence ratios (SIRs) and TMBs for a better understanding concerning the role of the immune system in transplant recipients with malignancies and the identification of opportunities to improve transplant safety.

## 2 METHODS

### 2.1 Data sources

A comprehensive and systemic search was conducted using network databases, including PubMed (update to February 2020), Web of Science (update to February 2020), EMBASE (1980 to February 2020), and Medline (1966 to February 2020). We used “lung transplantation” or “heart transplantation” combined with “cancer,” “neoplasm,” and “tumor” as well as their Medical Subject Headings (MeSH) terms. A manual search was conducted of the reference lists originating from retrieved review articles and conference abstracts. We contacted the author for supplemental data when important information was missing. Meanwhile, We evaluated all searched results according to the Preferred Reporting Items for Systematic Reviews and Meta‐Analyses (PRISMA) statement (Table S1).^17^ The protocol was registered in the Prospective Register of Systematic Reviews (PROSPERO CRD42020159599).

### 2.2 Study selection criteria

Studies pertaining to the affirmation of cancer risk among heart or lung transplant recipients were included if the following criteria were met: (1) population‐based cohort studies on heart or lung recipients; (2) study reported at least one site‐specific cancer risk (no matter positive or negative results) in heart or lung transplant recipients (3) SIRs and 95% confidence intervals (CIs) could be obtained or estimated from the article. Studies were excluded if they met any of the criteria below: (1) participants received other solid organ transplantations rather than lung or heart transplantation; (2) lack of available data with appropriate statistics; (3) studies were not published in English or duplicate publications.

### 2.3 Data extraction

Three authors (F.G., Z.H., R.W.) extracted the necessary data independently and any disagreements were resolved after discussion among the 3 investigators. The following information was recorded: the first author's name, reported outcomes, median of follow‐up duration, mean or median age of heart or lung transplant recipients, SIRs with their 95% CIs, country, study period, and publication year. In 2017, Chalmers et al.^18^ measured the distribution of TMB across a diverse cohort of 100,000 cancer cases through a targeted comprehensive genomic profiling (CGP) assay, and tested for association between somatic alterations and TMB in over 100 tumor types. We extracted the relevant TMBs directly when the malignancy of the included studies was found in Chalmers’ study. Otherwise, we took the average value of the related malignancies’ TMBs mentioned in the study (leukemia, melanoma, the cancer of cervix, brain, pancreas, liver, breast, prostate, colorectal, skin, lung, and bladder).

### 2.4 Quality assessment

The methodological quality of the selected studies was evaluated using criteria from the Newcastle Ottawa Scale (NOS) (Table 1), which included selection (4 items), comparability (1 item), and outcome (3 items).^19^ Any disagreement was resolved by consensus.

**TABLE 1:** Demographic details of the included studies

Study	Region	Number of transplant cases	Median/Mean age (years)	Median Follow‐u*p* Duration (years)	NOS scores	Reported outcomes
Heart transplantation						
Serraino et al. (2007)^25^	Italy	724	NA	6.2	7	All cancers, LC, Lung cancer, NHL, KS
Kellerman et al. (2009)^26^	USA	851	53.0	5.3	8	OC, LC, SC, CC, PC, PLC, Lung cancer, BC, Prostate cancer, KC, Bladder cancer, KS, Melanoma, Brain cancer
Collett et al. (2010)^27^	UK	3609	42.0	NA	7	All cancers, SCC, Lip cancer, OC, LC, AC, CC, Lung cancer, BCC, KC, HL, NHL, NMSC
Jiang et al. (2010)^28^	Canadian	1703	55.0	4.8	9	All cancers, OC, CC, PC, PLC, Lung cancer, BC, Prostate cancer, KC, Bladder cancer, NHL, Melanoma
Jensen et al. (2010)^29^	Denmark	459	50.0	NA	8	SCC, BCC, Melanoma
Engels et al., (2011)^30^	USA	17593	47.0	7.5	8	LC, Lung cancer, KC, NHL
Na et al. (2013)^7^	Australia	1518	47.0	5.2	8	All cancers, Lip cancer, OC, Esophagus cancer, LC, CC, Lung cancer, KC, HL, NHL, KS, NMSC, Melanoma, Brain cancer
Ohman et al. (2015)^33^	Sweden	437	42.9	5.7	8	Lip cancer, OC, KC, Bladder cancer
Safaeian et al. (2016)^34^	USA	22186	48.0	3.7	9	CC
Hortlund et al. (2017)^37^	Multiple (Denmark/Sweden)	506/894	45.5/45.5	8.8/8.8	8	All cancers, Lip cancer, KC, HL, NHL, NMSC
Jäämaa‐Holmberg et al. (2019)^41^	Finland	479	55.0	9.4	9	All cancers, SCC, Lip cancer, OC, Esophagus cancer, SC, CC, Lung cancer, Prostate cancer, KC, Bladder cancer, HL, NHL, KS, BCC, Melanoma
O'Neill et al. (2019)^43^	Ireland	214	47.1	7.0	8	All cancers, SCC, AC, Prostate cancer CC, Lung cancer, BC, NHL, BCC
Lung cancer transplantation						
Jensen et al. (2010)^28^	Denmark	384	53.0	5.0	8	HL, BCC, Melanoma
Collett et al. (2010)^27^	UK	2058	42.0	NA	7	All cancers, SCC, OC, LC, Lung cancer, BC, KC, HL, NHL, KS, NMSC
Engels et al. (2011)^30^	USA	7013	47.0	7.5	8	LC, CC, Lung cancer, KC, PTLD
Krynitz et al. (2013)^31^	Sweden	1012	50.0	5.0	8	All cancers, SCC, Lip cancer, OC, LC, SC, CC, PLC, Lung cancer, BC, Cervix cancer, VVC, Prostate cancer, Bladder cancer, HL, Leukemia, Skin cancer, Melanoma, Brain cancer, TC
Na et al. (2013)^7^	Australia	1200	47.0	5.2	8	All cancers, CC, Lung cancer, NHL, NMSC
Morton et al. (2014)^32^	USA	8543	57.0	10.0	8	HL, Leukemia
Ohman et al. (2015)^33^	Sweden	359	52.0	3.2	8	Lip cancer
Hortlund et al. (2017)^37^	Multiple (Denmark/Sweden)	471/584	45.5/45.5	8.8/8.8	8	All cancers, Lip cancer, NMSC
Ekstrom et al. (2017)^35^	Sweden	331	55.4	2.8	9	All cancers, SCC, LC, SC, CC, Lung cancer, BC, Cervix cancer, VVC, Prostate cancer, Bladder cancer, PTLD, NHL, Leukemia, Skin cancer
Fink et al. (2017)^36^	USA	1681	57.0	3.7	7	All cancers, CC, KC, NHL
Magruder et al. (2017)^38^	USA	18093	55.0	3.0	8	All cancers, OC, Esophagus cancer, LC, SC, CC, PLC. Lung cancer, BC, Cervix cancer, VVC, Prostate cancer, KC, Bladder cancer, PTLD, Leukemia, KS, non‐Melanoma, skin cancer, Brain cancer, TC
Rizvi et al. (2017)^39^	Norway	360	50.8	4.1	8	SCC
Tsai et al. (2019)^40^	China	1047	45.7	4.7	8	All cancers, SCC, OC, Esophagus cancer, LC, CC, Lung cancer, BC, Cervix cancer, Prostate cancer, Bladder cancer, HL, NMSC, Brain cancer, TC
O'Neill et al. (2019)^43^	Ireland	188	40.9	4.0	9	All cancers, SCC, CC, Lung cancer, Cervix cancer, VVC, Prostate cancer, PTLD, NHL, Skin cancer, BCC
Triplette et al. (2019)^44^	USA	8993	54.0	3.9	9	SCC, Lung cancer
Laprise et al. (2019)^42^	USA	12948	57.0	4.0	8	Lip cancer

Abbreviations: AC, Anus cancer; BC, Breast cancer; BCC, Basal cell carcinoma; CC, Colorectal cancer; HL, Hodgkin's lymphoma; KC, Kidney cancer; KS, Kaposi sarcoma; LC, Lung cancer; NHL, non‐Hodgkin's lymphoma; NMSC, non‐Melanoma skin cancer; OC, Oral cancer; PC, Pancreas cancer; PLC, Pharynx and larynx cance; PTLD, Posttransplant lymphoproliferative disorders; SC, Stomach cancer; TC, Thyroid cancer; VVC, Vulva and vagina cancer.

### 2.5 Statistical analysis

We examined cancer risks in heart or lung transplant recipients on the basis of the SIRs and their 95% CIs published in each study. A random‐effects model was adopted to calculate SIRs and 95% CIs^20^, ^21^ for heart or lung transplant recipients versus the general population. The synthesized SIRs were classified into 7 modules by anatomical site or histology: all cancers, digestive system, respiratory system, reproductive and urinary systems, lymphatic and hematological systems, integumentary system, and neurological system. We conducted subgroup analyses to investigate sources of heterogeneity and sensitivity analyses to explore whether any study had a large influence on the pooled‐effect estimates. Subgroup analysis based on age (50 years or older, <50 years) and region (Europe, Asia, North America, Oceania) was carried out. Sensitivity analysis was conducted by consecutive exclusion of each study. Heterogeneity was assessed using Cochran's *Q* test and the *I* ^2^ statistic; we defined statistical heterogeneity as noteworthy if an *I* ^2^ statistic ≥50%.^22^ Funnel plot tests, Egger's test,^23^ and Begg's test^24^ were utilized to appraise the publication bias. The population sizes of heart or lung transplantation were used as weights in the WLR to analyze the association and calculate the correlation coefficients between TMBs and SIRs in multiple‐site cancers. Because both TMBs and SIRs were not normally distributed, we took the logarithm of each and compared them. All statistical manipulation was carried out by Stata software (version 15, StataCorp). All *p*‐values were 2‐tailed; statistical significance was considered as *p*‐value <0.05.

## 3 RESULTS

### 3.1 Study selection

**FIGURE 1:**
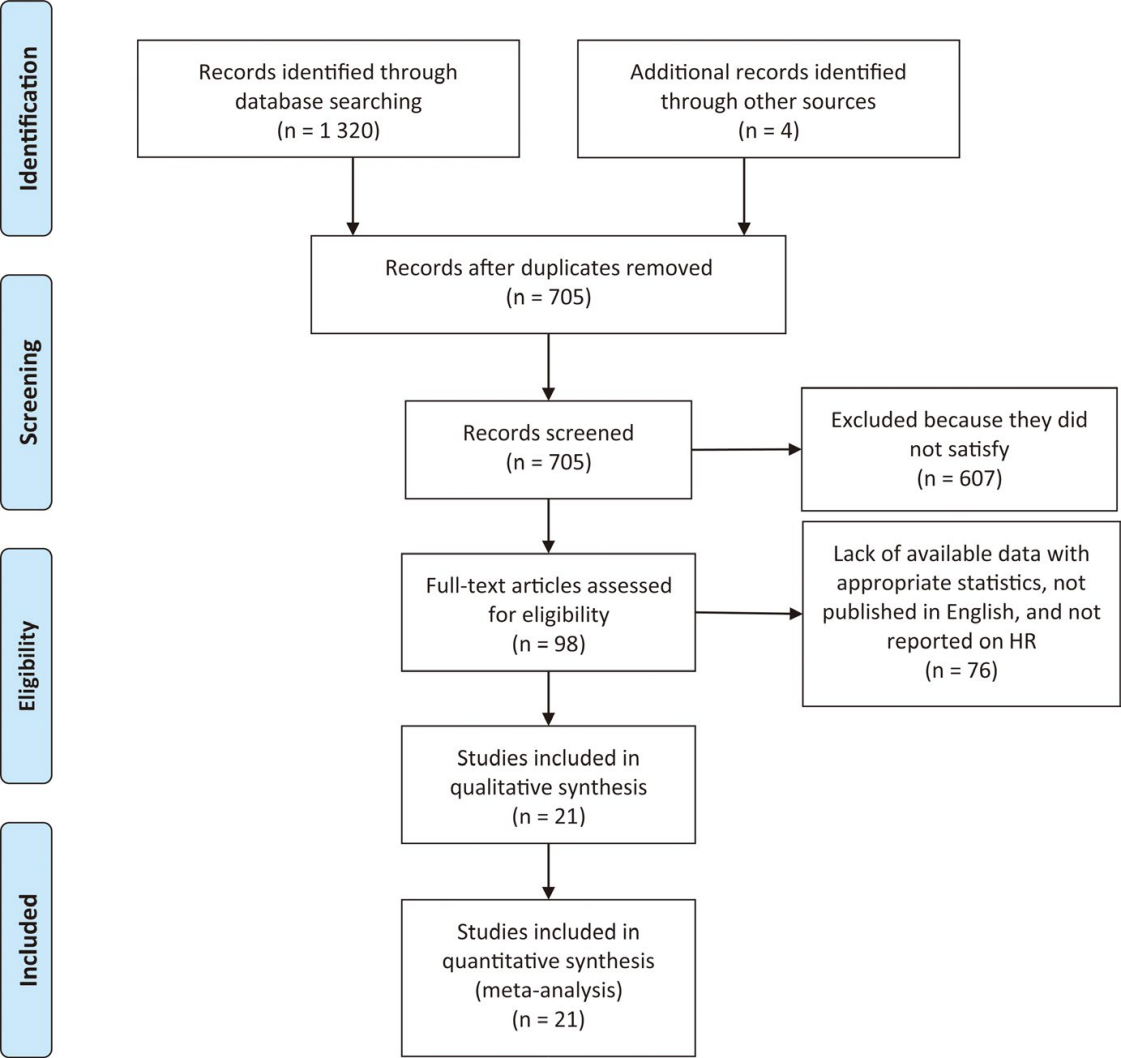
PRISMA diagram of study selection

In total, 1324 citations met the search criteria. After elimination of 619 duplicates, 705 underwent title and abstract screening. The full text of 98 articles was examined. Finally, 21^7^, ^25^, ^26^, ^27^, ^28^, ^29^, ^30^, ^31^, ^32^, ^33^, ^34^, ^35^, ^36^, ^37^, ^38^, ^39^, ^40^, ^41^, ^42^, ^43^, ^44^ of them met the inclusion criteria for our meta‐analysis (Figure 1).

### 3.2 Study characteristics

All 21 studies were prospective cohort studies. Table 1 shows the demographic details of the included studies. In heart or lung transplantation, 51,173 and 65,265 recipients were followed up for a total of 259,913.2 and 341,417.5 person‐years, with a median follow‐up time of 6.4 (range: 3.7–9.4 years) and 5.2 (range: 2.8–10.0 years) years, respectively. The mean or median age of heart and lung transplant recipients was 48.4 (range: 42.0–55.0) and 50.3 (range: 40.9–57.0) years old with organ transplantation, respectively.

### 3.3 Cancer risk in transplant recipients

A pooled analysis of 21 studies on the risk of various cancers in heart or lung transplant recipients is presented in Table 2. Furthermore, Figures S1–S4 show the forest plot for cancer at different sites. Both heart and lung transplant recipients were at a higher risk of all cancers, with a 3.13‐fold higher cancer risk [SIR: 3.13; 95% CI: 2.38–4.13; *p* < 0.001] and a 4.28‐fold higher cancer risk [SIR: 4.28; 95% CI: 3.18–5.77; *p* < 0.001], respectively. As for system‐specific cancers, among heart transplant recipients, the most common five systems were integumentary system [SIR: 22.86; 95% CI: 15.32–34.11; *p* < 0.001], lymphatic and hematological systems [SIR: 12.65; 95% CI: 8.58–18.94; *p* < 0.001], reproductive and urinary systems [SIR: 2.57; 95% CI: 1.85–3.56; *p* < 0.001], respiratory system [SIR: 2.43; 95% CI: 2.04–2.89; *p* < 0.001], digestive system [SIR: 1.48; 95% CI: 1.11–1.96; *p* = 0.007]. The most common five systems with the highest increased risk in lung transplant recipients were lymphatic and hematological systems [SIR: 14.24; 95% CI: 9.69–20.95; *p* < 0.001], integumentary system [SIR: 12.26; 95% CI: 7.03–22.81; *p* < 0.001], respiratory system [SIR: 5.90; 95% CI: 4.66–88.03; *p* < 0.001], digestive system [SIR: 3.49; 95% CI: 2.00–6.08; *p* < 0.001], reproductive and urinary systems [SIR: 1.96; 95% CI: 1.40–2.74; *p* < 0.001]. The results of sensitivity analyses are listed in Figures S5–S6, indicating that the omission of any single study did not result in a significant difference of the pooled results, except for cancer of colorectal, anus, liver, respiratory system, and reproductive and urinary systems in heart transplant recipients and cancer of esophagus, stomach, liver, pharynx and larynx, breast, cervix, vulva and vagina, kidney, bladder, basal cell carcinoma, and non‐melanoma in lung transplant recipients. However, the variable findings may be attributed to the limited number of included cohorts or the effects of heart or lung transplantation.

**TABLE 2:** SIRs of all cancers and specific cancer types by anatomical site or histology among heart or lung transplant recipients

Site	Heart					Lung				
	N	SIR (95% Cl)	*p*‐value	*I*‐square	*P*_heterogeneity_	*N*	SIR (95% Cl)	*p*‐value	*I*‐square	*P*_heterogeneity_
All cancers	8	3.13 (2.38, 4.13)	**<0.001**	96.2%	<0.001	10	4.28 (3.18, 5.77)	**<0.001**	95.8%	<0.001
Digestive system	20	1.48 (1.11, 1.96)	**0.007**	43.9%	0.019	19	3.49 (2.00, 6.08)	**<0.001**	92.1%	<0.001
Esophagus	2	1.73 (0.44, 6.89)	0.435	0.0%	0.812	2	3.26 (1.16, 9.12)	**0.025**	12.5%	0.285
Stomach	2	1.66 (0.37, 7.35)	0.506	0.0%	0.715	3	4.76 (1.58, 14.31)	**0.005**	58.8%	0.088
Colorectal	7	1.16 (0.84, 1.60)	0.371	51.7%	0.053	8	3.38 (1.44, 7.92)	**0.005**	96.0%	<0.001
Anus	2	8.49 (2.63, 27.39)	**<0.001**	0%	0.667	—	—	—	—	—
Liver	5	1.40 (0.89, 2.20)	0.149	0.0%	0.421	6	3.37 (1.23, 9.24)	**0.018**	70.9%	0.004
Pancreas	2	2.62 (0.91, 7.55)	0.075	0.0%	0.446	—	—	—	—	—
Respiratory system	16	2.90 (2.31, 3.64)	**<0.001**	61.4%	0.001	15	5.19 (4.04, 6.68)	**<0.001**	76.5%	<0.001
Pharynx and larynx	2	2.24 (0.67, 7.49)	0.190	0.0%	0.712	2	4.54 (1.58, 14.31)	**0.005**	0.0%	0.967
Lung	8	2.42 (2.01, 2.93)	**<0.001**	50.3%	0.050	9	5.97 (4.66, 7.66)	**<0.001**	80.4%	<0.001
Oral cavity	6	6.48 (3.40, 12.37)	**<0.001**	46.7%	0.095	4	2.34 (0.88, 6.25)	0.090	58.4%	0.065
Reproductive and urinary systems	20	2.57 (1.85, 3.56)	**<0.001**	19.9%	0.290	27	1.96 (1.40, 2.74)	**<0.001**	72.0%	0.770
Breast	3	1.72 (0.69, 4.27)	0.244	0.0%	0.705	5	0.77 (0.58, 1.03)	0.077	23.8%	0.283
Cervix	—	—	—	—	—	5	3.02 (1.07, 8.49)	**0.036**	64.5%	0.024
Vulva and vagina	—	—	—	—	—	4	10.16 (3.50, 29.50)	**<0.001**	0.0%	0.883
Prostate	4	1.29 (0.97, 1.71)	0.075	0.0%	0.924	5	1.25 (0.87, 1.81)	0.230	15.8%	0.314
Kidney	9	4.29 (2.90, 6.36)	**<0.001**	65.6%	0.003	4	2.71 (1.30, 5.64)	**0.008**	54.7%	0.085
Bladder	4	1.86 (0.83, 4.16)	0.129	19.9%	0.290	4	2.84 (1.81, 4.44)	**<0.001**	0.0%	0.770
Lymphatic and hematological systems	12	12.75 (8.58, 18.94)	**<0.001**	91.5%	<0.001	19	14.24 (9.69, 20.95)	**<0.001**	88.8%	<0.001
PTLD^a^	—	—	—	—	—	4	17.95 (15.33, 21.02)	**<0.001**	0.0%	0.607
Hodgkin's lymphoma	4	11.64 (6.50, 20.87)	**<0.001**	0.0%	0.912	5	8.83 (2.81, 27.69)	**<0.001**	91.1%	<0.001
non‐Hodgkin's lymphoma	8	13.00 (8.21, 20.59)	**<0.001**	94.6%	<0.001	6	29.62 (19.07, 46.03)	**<0.001**	82.1%	<0.001
Leukemia	—	—	—	—	—	4	2.15 (1.10, 4.17)	**0.024**	0.0%	<0.001
Integumentary system	27	22.86 (15.32, 34.11)	**<0.001**	96.9%	<0.001	27	12.66 (7.03, 22.81)	**<0.001**	98.5%	<0.001
Skin cancer	—	—	—	—	—	3	28.83 (9.44, 88.03)	**<0.001**	98.1%	<0.001
Kaposi sarcoma	4	112.76 (62.25, 204.25)	**<0.001**	0.0%	0.474	2	9.64 (1.26, 73.91)	**0.029**	0.0%	0.971
Lip	6	49.92 (29.26, 85.19)	**<0.001**	75.1%	0.001	5	29.15 (7.10, 119.72)	**<0.001**	95.6%	<0.001
Squamous cell carcinoma	4	55.54 (28.27, 109.13)	**<0.001**	95.5%	<0.001	7	10.65 (1.81, 62.54)	**0.009**	99.4%	<0.001
Basal cell carcinoma	4	7.74 (5.67, 10.58)	**<0.001**	70.3%	0.018	2	6.08 (3.02, 12.21)	**<0.001**	44.1%	0.181
non‐Melanoma skin cancer	4	39.31 (17.74, 87.09)	**<0.001**	98.1%	<0.001	6	10.72 (3.76, 30.59)	**<0.001**	97.5%	0.000
Melanoma	5	3.06 (2.23, 4.19)	**<0.001**	0.0%	0.981	2	2.43 (0.85, 6.97)	0.098	0.0%	0.955
Neurological	2	2.28 (0.69, 7.52)	0.178	0.0%	0.403	6	2.18 (1.22, 3.92)	**0.009**	16.5%	0.308
Brain	2	2.28 (0.69, 7.52)	0.178	0.0%	0.403	3	2.12 (0.93, 4.83)	0.075	0.0%	0.915
Thyroid	—	—	—	—	—	3	2.69 (0.75, 9.74)	0.131	65.5%	0.055

Note

An SIR >1 suggests that the cancer risk is higher than that of the ordinary population; The *p*‐values less than 0.05 are in bold.

Abbreviations: CI, Confidence interval; N, Number of studies; PTLD, Posttransplant lymphoproliferative disorders; SIR, Standardized incidence ratio.

^a^ PTLD is not a separate category from Non‐Hodgkin lymphoma or Hodgkin lymphoma.

### 3.4 Correlation between SIRs and TMBs

**FIGURE 2:**
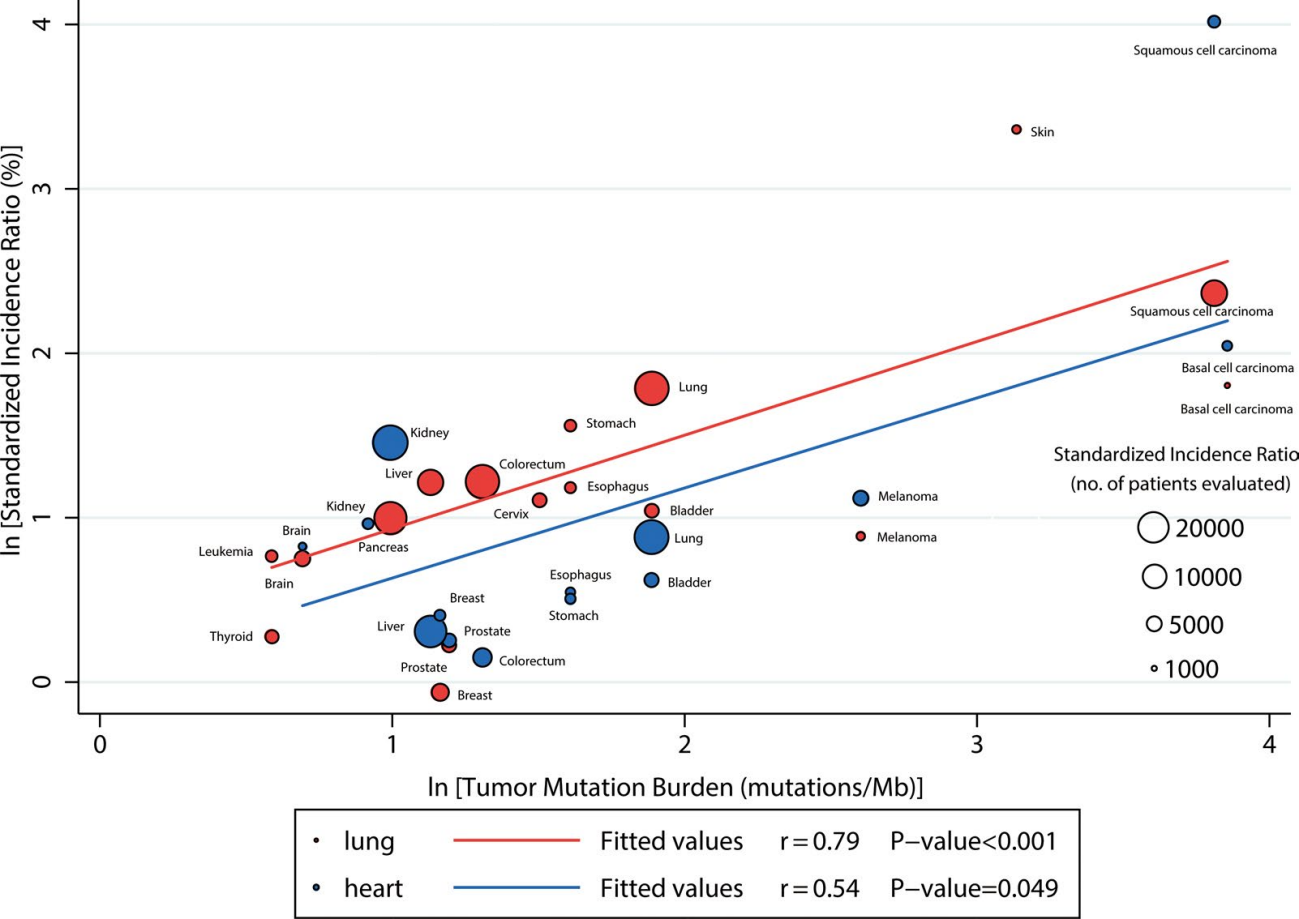
Correlation between Tumor Mutational Burdens and Standardized Incidence Ratios in heart or lung transplant recipients. The number of transplant recipients who were analyzed for the SIR is shown for each tumor type (size of the circle). Data on the x and y axis are shown on a logarithmic scale

In heart or lung transplant recipients, we observed a significant correlation between TMBs and SIRs (heart transplantation: *p* = 0.049; lung transplantation: *p* < 0.001). The correlation coefficients between SIRs and TMBs (heart transplantation: 0.54 and lung transplantation: 0.79) suggested that 29% and 63% of the differences in SIRs across cancer types might be explained by the TMBs, respectively (Figure 2).

### 3.5 Subgroup analyses

The results of our subgroup analyses are presented in Table 3. Furthermore, Figures S7–S10 show the forest plot for cancer at different sites. We performed subgroup analyses according to age (50 years or older, <50 years), and region (Europe, Asia, North America, Oceania). First, we found that heart transplant recipients older than 50 years were at higher risk for cancers than those younger than 50 years, with the exception of lung cancer. Second, lung transplant recipients had significantly higher risks of lung and liver cancers in Europe than in other regions. Third, our study denoted that lung transplant recipients were at the highest risk for all cancers in South America. Fourth, most malignancies were at increased risks worldwide in transplant recipients, while a few tumors were at decreased risk. It may be associated with the different susceptibilities of the malignancies in different regions. Subgroup analysis showed that the age and region of transplant recipients are not the source of heterogeneity.

**TABLE 3:** SIRs of all cancers and specific cancer types among heart or lung transplant recipients in subgroup analysis

Site	Heart					Lung				
	N	SIR (95% Cl)	*p*‐value	*I*‐square	*P*_heterogeneity_	*N*	SIR (95% Cl)	*p*‐value	*I*‐square	*P*_heterogeneity_
Age group										
<50 yr	34	3.68 (2.72, 4.97)	**<0.001**	97.4%	<0.001	46	4.37 (3.01, 6.35)	**<0.001**	97.9%	<0.001
All cancers	5	2.95 (2.23, 3.90)	**<0.001**	93.8%	<0.001	7	3.52 (2.80, 4.41)	**<0.001**	85.6%	<0.001
Squamous cell carcinoma	2	46.24 (8.08, 254.66)	**<0.001**	97.7%	<0.001	3	4.28 (0.48, 37.80)	0.191	95.4%	<0.001
Oral cavity	3	5.02 (2.59, 9.74)	**<0.001**	0.0%	0.525	3	3.14 (0.80, 12.33)	0.102	61.7%	0.074
Colorectal	4	0.99 (0.85, 1.17)	0.942	0.0%	0.665	5	1.49 (1.09, 2.04)	**0.014**	23.4%	0.265
Liver	3	1.10 (0.65, 1.85)	0.725	0.0%	0.806	4	2.23 (0.55, 9.09)	0.264	72.1%	0.013
Lung	4	2.51 (2.18, 2.89)	**<0.001**	18.1%	0.300	6	5.12 (3.99, 6.57)	**<0.001**	48.7%	0.083
Cervix	—	—	—	—	—	3	3.86 (0.92, 16.26)	0.065	42.4%	0.176
Prostate	—	—	—	—	—	3	1.64 (0.93, 2.89)	0.089	0.0%	0.369
Kidney	6	4.07 (2.70, 6.12)	**<0.001**	57.8%	0.037	2	1.63 (0.81, 3.26)	0.171	0.0%	0.586
Bladder	—	—	—	—	—	2	2.00 (0.77, 5.19)	0.153	0.0%	0.504
PTLD^a^	—	—	—	—	—	2	18.62 (15.57, 22.25)	**<0.001**	0.0%	0.519
non‐Hodgkin's lymphoma	5	8.89 (5.05, 15.66)	**<0.001**	93.9%	<0.001	4	22.82 (17.01, 30.63)	**<0.001**	40.7%	0.504
non‐Melanoma skin cancer		—	—	—	—	4	20.23 (7.30, 56.08)	**<0.001**	97.4%	<0.001
Melanoma	2	3.00 (2.06, 4.37)	**<0.001**	0.0%	0.660	—	—	—		
≥50 yr	25	4.56 (2.57, 8.08)	**<0.001**	98.0%	<0.001	26	7.25 (3.70, 14.10)	**<0.001**	99.3%	<0.001
All cancers	2	4.03 (1.84, 8.82)	**<0.001**	98.3%	<0.001	3	6.83 (2.93, 16.93)	**<0.001**	98.7%	<0.001
Squamous cell carcinoma	2	67.14 (41.32, 109.09)	**<0.001**	88.3%	0.003	4	20.92 (1.98, 220.51)	**0.011**	99.6%	<0.001
Oral cavity	3	8.37 (2.43, 28.91)	**0.001**	74.1%	0.021			—		
Colorectal	3	1.35 (0.44, 4.12)	0.600	76.7%	0.014	3	8.25 (1.71, 39.74)	**0.009**	96.8%	<0.001
Liver	1	3.30 (0.61, 18.00)	0.522	—	—	2	6.62 (1.10, 40.04)	**0.039**	79.6%	0.027
Lung	3	2.05 (1.00, 4.17)	**0.049**	79.3%	0.008	3	8.10 (4.42, 14.85)	**<0.001**	93.5%	<0.001
Cervix	—	—	—	—	—	2	2.44 (0.40, 14.68)	0.334	85.8%	<0.001
Prostate	—	—	—	—	—	2	1.04 (0.76, 1.42)	0.817	0.0%	0.352
Kidney	3	4.32 (1.61, 11.60)	**0.004**	71.5%	0.030	2	4.48 (0.97, 20.60)	0.054	77.6%	0.035
Bladder	3	1.69 (0.61, 4.65)	0.313	46.6%	0.154	2	3.13 (1.89, 5.21)	**<0.001**	0.0%	0.880
PTLD^a^	—	—	—	—	—	2	17.95 (15.33, 21.02)	**<0.001**	0.0%	0.607
non‐Hodgkin's lymphoma	2	23.78 (19.30, 29.29)	**<0.001**	0.0%	0.572	2	52.19 (33.79, 80.63)	**<0.001**	43.2%	0.185
non‐Melanoma skin cancer	—	—	—	—	—	1	2.19 (1.31, 3.69)	**0.004‐**		
Melanoma	3	3.20 (1.81, 8.82)	**<0.001**	0.0%	0.910	—	—	—		
Region group		—	—		—					
South America	15	2.74 (1.66, 4.50)	**<0.001**	97.8%	<0.001	18	4.86 (3.16, 7.49)	**<0.001**	98.6%	<0.001
All cancers	1	2.70 (2.30, 3.20)	**<0.001**	—	—	4	5.07 (3.21, 8.02)	**<0.001**	96.5%	<0.001
Squamous cell carcinoma	—	—	—	—	—	1	8.10 (6.30, 10.30)	**<0.001**		
Oral cavity	2	4.42 (2.46, 7.93)	**<0.001**	0.0%	0.805	—	—	—		
Colorectal	3	0.95 (0.79, 1.13)	0.553	0.0%	0.937	3	3.88 (0.98, 15.41)	0.054	98.3%	<0.001
Liver	2	1.39 (0.50, 3.80)	0.527	39.2%	0.200	2	2.46 (1.20, 5.05)	**0.014**	0.0%	0.667
Lung	3	1.95 (1.20, 3.15)	**0.007**	75.4%	0.017	3	5.67 (4.68, 6.87)	**<0.001**	69.4%	0.038
Kidney	1	2.88 (2.33, 3.55)	**<0.001**	—	—	3	2.84 (1.16, 6.98)	**0.023**	69.8%	0.036
PTLD^a^	—	—	—	—	—	2	17.42 (13.69, 22.17)	**<0.001**	30.3%	0.231
non‐Hodgkin's lymphoma	2	13.21 (4.63, 37.68)	**<0.001**	98.1%	<0.001	1	61.80 (43.52, 87.75)‐	**<0.001**		
non‐Melanoma skin cancer	—	—	—	—	—	1	2.19 (1.31, 3.71)	**0.003**		
Melanoma	2	2.95 (1.47, 5.92)	**0.002**	0.0%	0.886	—	—	—		
Europe	37	7.38 (4.86, 11.21)	**<0.001**	98.8%	<0.001	31	9.80 (5.24, 18.32)	**<0.001**	98.8%	<0.001
All cancers	6	3.29 (2.30, 4.72)	**<0.001**	96.8%	<0.001	4	4.50 (2.47, 8.20)	**<0.001**	96.5%	<0.001
Squamous cell carcinoma	4	55.54 (28.27, 109.13)	**<0.001**	95.5%	<0.001	5	15.70 (1.52, 161.03)	**0.021**	99.2%	<0.001
Oral cavity	3	10.06 (3.28, 4.72)	**<0.001**	65.8%	0.053	2	6.42 (2.24, 18.34)	**0.001**	0.0%	0.714
Colorectal	3	1.87 (0.83, 4.21)	**0.034**	70.5%	0.034	3	3.88 (0.73, 20.77)	0.113	77.4%	0.012
Liver	2	2.15 (0.87, 5.32)	0.366	0.0%	0.366	3	9.96 (4.37, 22.71)	**<0.001**	0.0%	0.375
Lung	4	2.76 (2.04, 3.75)	**<0.001**	43.9%	0.148	4	8.73 (4.39, 17.34)	**<0.001**	82.2%	0.001
Kidney	5	6.35 (4.21, 9.60)	**<0.001**	33.5%	0.198	1	2.50 (0.46, 13.69)	0.632		
PTLD^a^	—	—	—	—	—	2	17.95 (15.33, 21.02)	**<0.001**	0.0%	0.607
non‐Hodgkin's lymphoma	5	16.70 (11.11, 24.97)	**<0.001**	71.9%	0.007	4	27.96 (22.29, 35.08)	**<0.001**	0.0%	0.403
non‐Melanoma skin cancer	3	41.52 (16.13, 106.85)	**<0.001**	98.7%	<0.001	3	39.99 (13.70, 108.75)	**<0.001**	97.5%	<0.001
Melanoma	2	3.38 (1.35, 8.43)	**0.009**	0.0%	<0.001	2	2.43 (0.85, 6.97)	0.098	0.0%	0.955
Asia						7	1.79 (1.26, 2.56)	**0.001**	29.5%	0.203
All cancers	—	—	—	—	—	1	1.65 (1.21, 2.24)	**0.001**	—	—
Squamous cell carcinoma	—	—	—	—	—	1	1.99 (0.95, 4.17)	0.068	—	—
Oral cavity	—	—	—	—	—	—	—	—	—	—
Colorectal	—	—	—	—	—	1	1.99 (0.95, 4.17)	0.843	—	—
Liver	—	—	—	—	—	1	0.21 (0.03, 1.49)	0.068	—	—
Lung	—	—	—	—	—	1	2.29 (1.52, 5.61)	0.117	—	—
Kidney	—	—	—	—	—	1	2.29 (1.52, 5.61)	**0.001**	—	—
non‐Hodgkin's lymphoma	—	—	—	—	—	—	—	—	—	—
non‐Melanoma skin cancer	—	—	—	—	—	1	2.98 (0.42, 21.20)	0.275	—	—
Melanoma	—	—	—	—	—	—	—	—	—	—
Oceania	8	2.61 (1.64, 4.13)	**<0.001**	88.5%	<0.001	5	4.42 (2.28, 8.59)	**<0.001**	91.7%	<0.001
All cancers	1	2.64 (2.32, 2.98)	**<0.001**	—	—	1	4.28 (3.49, 5.19)	**<0.001**	—	—
Squamous cell carcinoma	—	—	—	—	—	—	—	—	—	‐
Oral cavity	1	1.41 (0.04, 7.88)	0.799	—	—	—	—	—	—	—
Colorectal	1	0.99 (0.54, 1.63)	0.972	—	—	1	2.58 (1.12, 5.09)	**0.014**	—	—
Liver	1	1.85 (0.22, 6.69)	0.480	—	—	—	—	—	—	—
Lung	1	2.18 (1.39, 3.22)	**<0.001**	—	—	1	3.82 (1.65, 3.53)	**<0.001**	—	—
Kidney	1	2.36 (0.87, 5.14)	0.058	—	—	—	—	—	—	—
non‐Hodgkin's lymphoma	1	7.80 (5.71, 10.41)	**<0.001**	—	—	1	16.8 (11.1, 24.4)	**<0.001**	—	—
non‐Melanoma skin cancer	—	—	—	—	—	1	1.64 (0.53, 3.83)	0.327	—	—
Melanoma	1	3.04 (2.03, 4.36)	**<0.001**	—	—	—	—	—	—	—

Note

An SIR >1 suggests that the cancer risk is higher than that of the ordinary population; The *p*‐values less than 0.05 are in bold.

Abbreviations: CI, Confidence interval; N, Number of studies; PTLD, Posttransplant lymphoproliferative disorders; SIR, Standardized incidence ratio; yr, years old.

^a^ PTLD is not a separate category from Non‐Hodgkin lymphoma or Hodgkin lymphoma.

### 3.6 Publication bias analysis

Significant heterogeneity existed in the pooled analyses. With the limited information available, we were unable to detect any source leading to substantial heterogeneity. Furthermore, Egger's and Begg's test results showed no evidence of publication bias for all cancers analyzed in heart or lung transplant recipients (Figures S11–S12).

## 4 DISCUSSION

Our large‐scale quantitative study included 116,438 transplant recipients (51,173 heart transplant recipients and 65,265 lung transplant recipients) from 29 cohorts (12 cohorts for heart transplantation and 17 cohorts for lung transplantation). In terms of our results, we found that heart and lung transplant populations had significantly increased risks of cancer compared with the general population. Moreover, the incidences of various malignancies we observed correlate closely with data previously reported for a variety of post‐transplantation patient populations.

In 2011, Engels et al.^30^ conducted a large cohort study to calculate the risk of cancers in solid organ transplant recipients. They found that the Non‐Hodgkin's lymphoma (NHL) incidence was the highest in lung recipients, which is consistent with the results we observed. However, they reported that the risk of breast and prostate cancer were decreased in solid organ transplant recipients, we did not find the association in heart or lung transplantation. Overall, it does appear that heart or lung transplant patients remain more vulnerable to malignancy than the overall solid organ transplant population [The SIR of heart and lung transplant patients are 3.13 (2.38, 4.13) and 4.28 (3.18, 5.77), respectively, versus 2.10 (2.06–2.14) for all solid organ transplant recipients]. Heo et al.^45^ reported the cancer risk among renal transplant recipients. Similarly, NHL also showed a higher risk [SIR: 28.64 (7.70–73.32)] in renal transplantation. We found that the incidence of kidney cancer was higher in renal transplantation than in heart or lung transplantation. [The SIR of heart and lung transplant patients are 4.29 (2.90, 6.36) and 2.71 (1.30, 5.64), respectively, versus 16.31 (7.44–30.95) for renal transplant recipients]. Furthermore, the findings of our subgroup analyses indicated that the risk of certain cancers (e.g., lung cancer, liver cancer) varied by regions and ages, suggesting the presence of ethnicity‐based and age‐based differences. As the most common cause of mortality in patients with solid organ transplant,^46^ we observed that the incidence of posttransplant lymphoproliferative disorders (PTLD) increased significantly in lung transplantation and the rate not influenced by age and region.

Several mechanisms may explain the increased cancer risk for heart or lung transplant recipients. Both viral and nonviral factors are involved in cancer progression after heart or lung transplantation. Infection with the hepatitis C and hepatitis B virus are considered risk factors for liver cancer, while Epstein‐Barr (EB) virus infection may be associated with an increased risk of non‐Hodgkin's lymphoma.^47^, ^48^ Additionally, risks also increased for certain malignancies without established links to infections. The risk of few cancers (e.g., non‐melanoma skin cancer and lip cancer) are increased in HIV‐infected populations^12^, ^25^ which may reflect the loss of immune surveillance, activation of the immune system, or the effects of chronic inflammation. Transplant recipients have higher risks of colorectal and lip cancer than HIV‐infected individuals.^12^

Compared with heart transplantation, lung cancer risk was higher among lung recipients, perhaps due to the smoking‐related lung diseases (eg, chronic obstructive pulmonary disease) that could be the indication for a lung transplant, which leads to a worse prognosis in the transplant state. Moreover, lung cancer risk increased over time among lung recipients, suggesting a cumulative effect of transplantation.^30^ In lung recipients receiving single‐lung transplantation, most lung cancers occur in the other natural lung.^49^, ^50^ However, some cancers occurred in the first 6 months after transplantation may have cancers before surgery but delayed reports of cancer discovery in the explanted lung.^51^, ^52^ It could be the potential reasons for the difference in malignancy rates between heart transplantation and lung transplantation.

Long‐term use of immunosuppressive therapy is related to the increased incidence of cancer. Immunosuppression is possibly related to the direct damage of cells and cell repair systems.^53^, ^54^ Generally, immunosuppressants act by depleting T cells, leading to decreased acute rejection rates and increased graft survival.^55^ Immunosuppressants also have the ability to reduce immune surveillance, which facilitates the survival and proliferation of abnormal cells.^56^ In lung transplantation, the use of immunosuppressants is more intensive and the large amount of lymphoid tissue conveyed within the lung graft is the likely cause of the significantly increased risk of non‐Hodgkin's lymphoma in lung transplant recipients.^6^ In addition, the significant elevation of skin‐related malignancies (e.g., basal cell carcinoma and SCC) and cervical cancer in transplant recipients may be related to the increased susceptibility to human papillomavirus.^57^ Compared with 11% to 32% in normal skin, up to 90% of SCCs in solid organ transplantation recipients contain human papillomavirus DNA.^58^ Immunosuppressants have also shown the possibility to increase the risk of UV‐related carcinogenic effects.^59^, ^60^

TMB is a promising biomarker for predicting the response to immune checkpoint inhibitors of solid tumors.^61^ To some extent, TMB reflects the immunogenicity of the tumor. The higher the TMB of a certain cancer is, the more types of abnormal proteins are produced, which would be recognized as antigens, leading to a higher possibility of being recognized by the immune system. Therefore, when the immune system is normal, malignancies with a high TMB are less likely to grow. Immunosuppressive drugs lower the ability of immune surveillance of the immune system, leading to increased survival of high‐TMB malignancies, which supports the increased risk of cancer in transplant recipients. In 2019, D’Arcy et al.^62^ reported the survival after cancer diagnosis among solid organ transplant recipients. They found that for most cancers (e.g., melanoma, breast cancer, bladder cancer, colorectal cancer), the cancer‐specific mortality rate of transplant recipients was higher than the cancer patients, especially increase in melanoma, which may be due to the use of immunosuppressants leading to impaired immunity in transplant recipients. Furthermore, melanoma has a higher TMB than most other cancers according to Chalmers et al. study. These findings indicate that the use of immunosuppressants may be an important factor in promoting the occurrence and development of cancers in transplant recipients. The correlation coefficients between SIRs and TMBs suggested that 29% and 63% of the differences in SIRs across cancer types might be explained by the TMBs, respectively. However, as for the remaining 71% and 37%, we tend to believe that it might be explained by the following reasons: the different susceptibilities to different malignancies in transplant recipients, use of individualized doses of immunosuppressants, or the insufficient intensity of cancer screening in transplant recipients. The high correlation between the cancers’ SIRs and their TMBs in lung transplantation may be related to high immunosuppressive intensity in lung transplantation.^6^

There are several strengths to this study. First, to our knowledge, this is the first comprehensive meta‐analysis estimating the risk of each site‐specific cancer after heart or lung transplantation and exploring the relationship between corresponding SIRs and their TMBs. Second, the large sample size allowed us to quantitatively assess the impact of heart or lung transplantation on the risk of cancer at multiple sites, thus, our findings were more reliable than any individual study. Third, the SIRs were calculated across subgroups, which could assess the impact of heart or lung transplantation in different populations.

We acknowledge some limitations in regards to our meta‐analysis: first, heterogeneity between studies was high, which may be due to the following: (1) no detailed information on the smoking status,^63^ body mass index,^64^ alcohol use^65^ and immunosuppressants^66^ were available that allow us to perform an adjustment for these potential confounders; and (2) although all studies used the general population as reference, the matching criteria for studies in different countries may be different. Second, as there was no pre‐transplant disease data for heart or lung transplant recipients, we cannot determine whether this information would have an effect on heart or lung transplant recipients’ risk of developing cancer. However, this information was very important, which could be the source of bias, such as cystic fibrosis, a prime reason for lung transplantation, it also increases the incidence of gastrointestinal cancers regardless of whether the patient has a transplantation. Third, due to data limitations, we are unable to analyze different subtypes of tumors to explore the impact of tumor properties on the cancer risk of transplant recipients.

In conclusion, this meta‐analysis demonstrated that both heart and lung transplant recipients displayed a higher risk in site‐specific cancers and for most cancers, the cancer‐specific risk was higher in lung transplantation than heart transplantation. Moreover, the correlation between TMBs and SIRs in lung transplantation is higher, which may due to the high immunosuppressive intensity. Such associations can provide individualized guidance for clinicians in the detection of cancer among heart or lung transplantation recipients. In addition, we provided evidence that the risks of different cancers might be related to TMB, suggesting that the increased risks of post‐transplant cancers were attributed to the intervention of immunosuppression.

## CONFLICT OF INTEREST

The authors of this manuscript have no conflicts of interest to disclose.

## AUTHOR CONTRIBUTIONS

All authors contributed to the design of the study and to the drafting of the paper and have seen and approved the final version.

## Supporting information

Table S1‐Fig S1‐S12

## Data Availability

The raw data supporting the conclusions of this article will be made available by Jianxing He (drjianxing.he@gmail.com) for a period of 5 years after the publication date.
