# Supplementary material for: Cancer risk in heart or lung transplant recipients: A comprehensive analysis of 21 prospective cohorts
Source: Cancer Med. 2020 Oct 13;9(24):9595–610. doi: 10.1002/cam4.3525 (PMC7774758; doi:10.1002/cam4.3525)
Supplement: Supplementary file 1 — Table S1‐Fig S1‐S12 [file CAM4-9-9595-s001.pdf]

Cancer Risk in Heart or Lung Transplant Recipients: A Comprehensive Analysis of 21 Prospective Cohorts

Fan Ge, Caichen Li, Xin Xu, Zhenyu Huo, Runchen Wang, Yaokai Wen, Haoxin Peng, Xiangrong Wu, Hengrui Liang, Guilin Peng, Run Li, Danxia Huang, Ying Chen, Shan Xiong, Ran Zhong, Bo Cheng, Jianfu Li, Jianxing He, Wenhua Liang.

Supplementary materials

Table of Contents

		Page
**Table S1**	Checklist of the PRISMA extension	**3-5**
**Figure S1**	Forest plot for all cancers in heart transplantation	**6**
**Figure S2**	Forest plots for different systems cancers in heart transplantation	**7**
**Figure S3**	Forest plot for all cancers in lung transplantation	**8**
**Figure S4**	Forest plots for different systems cancers in lung transplantation	**9**
**Figure S5**	Sensitivity analysis for cancers in heart transplantation.	**10**
**Figure S6**	Sensitivity analysis for cancers in lung transplantation.	**11-12**
**Figure S7**	Forest plots for cancers in heart transplant recipients at different ages	**13**
**Figure S8**	Forest plots for cancers in heart transplant recipients at different regions	**14**
**Figure S9**	Forest plots for cancers in lung transplant recipients at different ages	**15**
**Figure S10**	Forest plots for cancers in lung transplant recipients at different regions	**16**
**Figure S11**	Funnel plots for cancers in heart transplantation.	**17**
**Figure S12**	Funnel plots for cancers in lung transplantation.	**18-19**

Section/topic	#	Checklist item	Reported on page #
**TITLE**			
Title	1	Identify the report as a systematic review, meta-analysis, or both.	1
**ABSTRACT**			
Structured summary	2	Provide a structured summary including, as applicable: background; objectives; data sources; study eligibility criteria, participants, and interventions; study appraisal and synthesis methods; results; limitations; conclusions and implications of key findings; systematic review registration number.	3
**INTRODUCTION**			
Rationale	3	Describe the rationale for the review in the context of what is already known.	4-5
Objectives	4	Provide an explicit statement of questions being addressed with reference to participants, interventions, comparisons, outcomes, and study design (PICOS).	4-5
**METHODS**			
Protocol and registration	5	Indicate if a review protocol exists, if and where it can be accessed (e.g., Web address), and, if available, provide registration information including registration number.	5
Eligibility criteria	6	Specify study characteristics (e.g., PICOS, length of follow-up) and report characteristics (e.g., years considered, language, publication status) used as criteria for eligibility, giving rationale.	5-6
Information sources	7	Describe all information sources (e.g., databases with dates of coverage, contact with study authors to identify additional studies) in the search and date last searched.	6
Search	8	Present full electronic search strategy for at least one database, including any limits used, such that it could be repeated.	5-6
Study selection	9	State the process for selecting studies (i.e., screening, eligibility, included in systematic review, and, if applicable, included in the meta-analysis).	5-6
Data collection process	10	Describe method of data extraction from reports (e.g., piloted forms, independently, in duplicate) and any processes for obtaining and confirming data from investigators.	6
Data items	11	List and define all variables for which data were sought (e.g., PICOS, funding sources) and any assumptions and simplifications made.	6-7
Risk of bias in individual studies	12	Describe methods used for assessing risk of bias of individual studies (including specification of whether this was done at the study or outcome level), and how this information is to be used in any data	6

		synthesis.	
Summary measures	13	State the principal summary measures (e.g., risk ratio, difference in means).	6-7
Synthesis of results	14	Describe the methods of handling data and combining results of studies, if done, including measures of consistency (e.g., I^2) for each meta-analysis.	6-7
Risk of bias across studies	15	Specify any assessment of risk of bias that may affect the cumulative evidence (e.g., publication bias, selective reporting within studies).	8-10
Additional analyses	16	Describe methods of additional analyses (e.g., sensitivity or subgroup analyses, meta-regression), if done, indicating which were pre-specified.	6-7
**RESULTS**			
Study selection	17	Give numbers of studies screened, assessed for eligibility, and included in the review, with reasons for exclusions at each stage, ideally with a flow diagram.	Figure 1
Study characteristics	18	For each study, present characteristics for which data were extracted (e.g., study size, PICOS, follow-up period) and provide the citations.	7-8
Risk of bias within studies	19	Present data on risk of bias of each study and, if available, any outcome level assessment (see item 12).	8-9 and Supplementary Materials page 7
Results of individual studies	20	For all outcomes considered (benefits or harms), present, for each study: (a) simple summary data for each intervention group (b) effect estimates and confidence intervals, ideally with a forest plot.	8-9 and Supplementary Materials page 6 and Table 2
Synthesis of results	21	Present results of each meta-analysis done, including confidence intervals and measures of consistency.	8-9 and Supplementary Materials page 6, 8, Table 2, and Table 3
Risk of bias across studies	22	Present results of any assessment of risk of bias across studies (see Item 15).	8-10 and Supplementary Materials page 7-9

Additional analysis	23	Give results of additional analyses, if done (e.g., sensitivity or subgroup analyses, meta-regression [see Item 16]).	8-9, Figure 2, Table2, and Supplementary Materials page 7-8
**DISCUSSION**			
Summary of evidence	24	Summarize the main findings including the strength of evidence for each main outcome; consider their relevance to key groups (e.g., healthcare providers, users, and policy makers).	10-13
Limitations	25	Discuss limitations at study and outcome level (e.g., risk of bias), and at review-level (e.g., incomplete retrieval of identified research, reporting bias).	10-13
Conclusions	26	Provide a general interpretation of the results in the context of other evidence, and implications for future research.	10-13
**FUNDING**			
Funding	27	Describe sources of funding for the systematic review and other support (e.g., supply of data); role of funders for the systematic review.	2

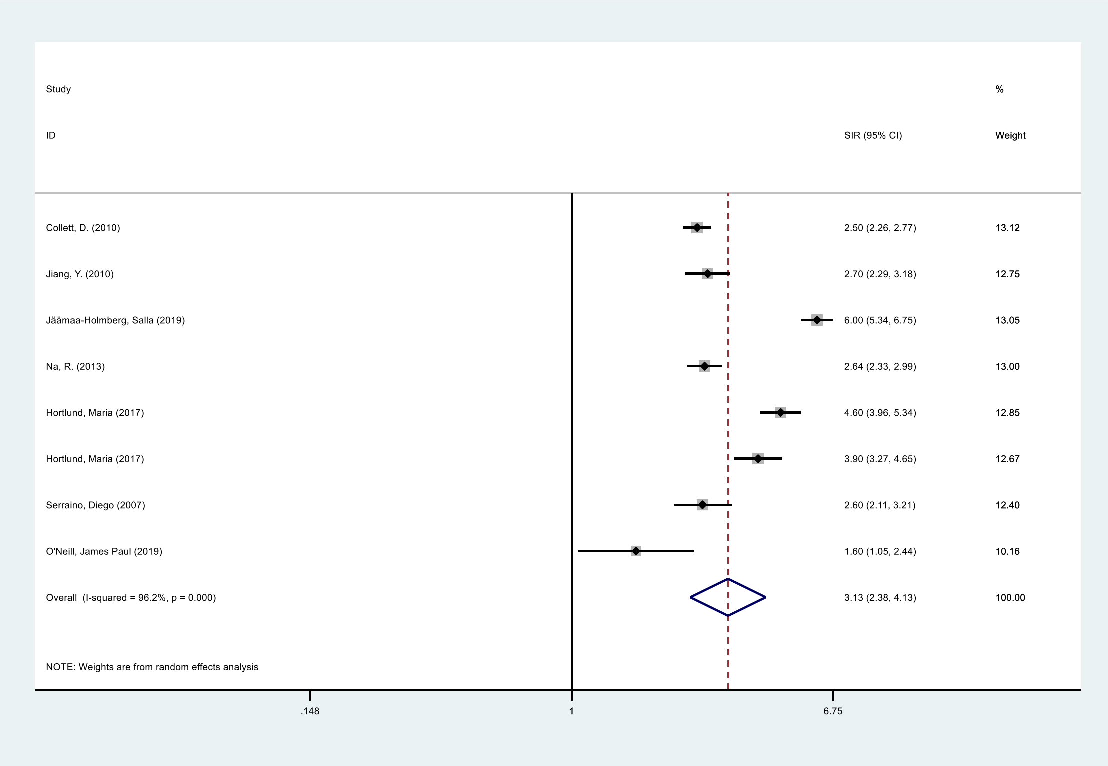

Figure S1: Forest plot for all cancers in heart transplantation.

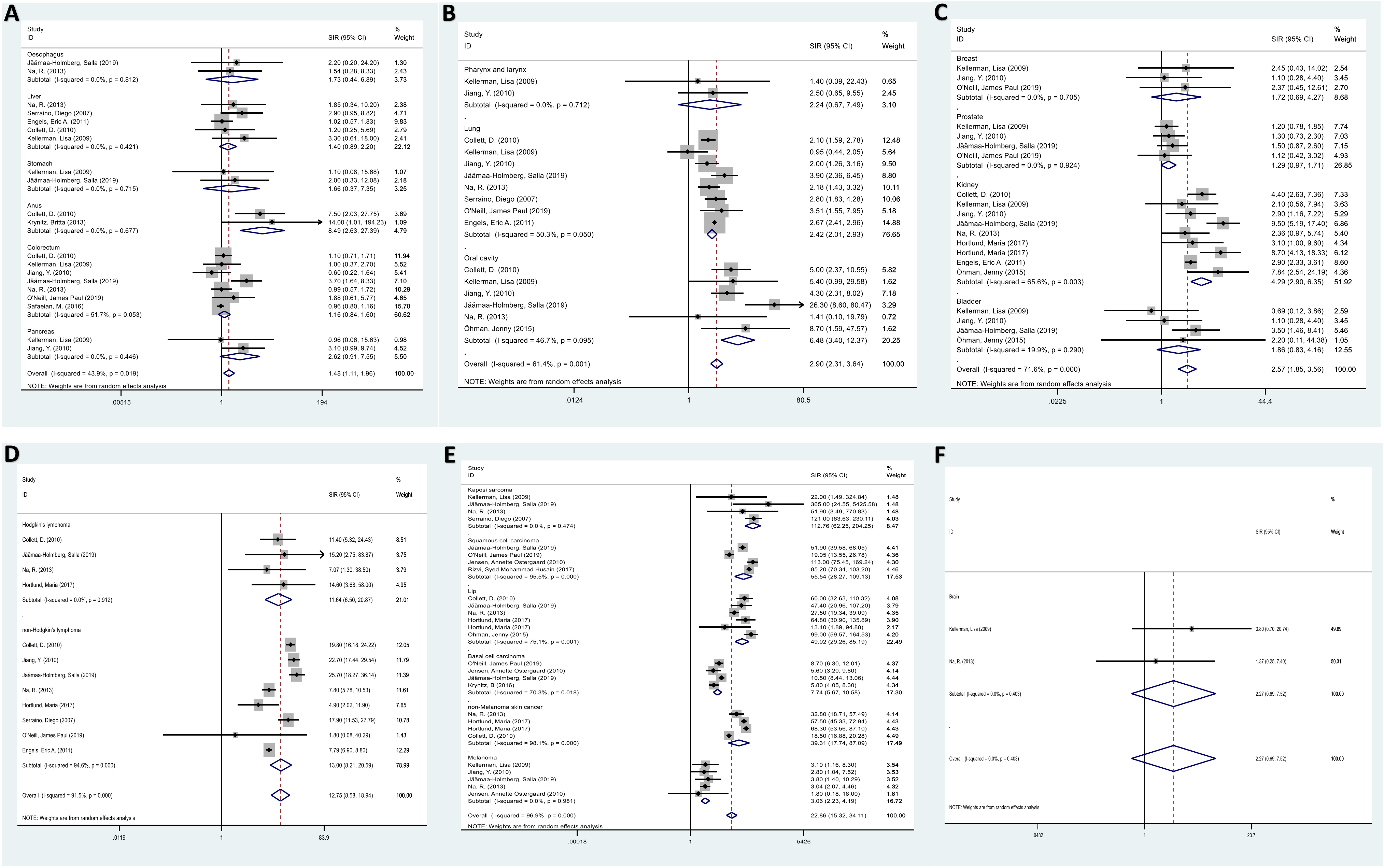

Figure S2: Forest plots for different systems cancers in heart transplantation. Digestive system (A), respiratory system (B), reproductive and urinary systems(C), lymphatic and hematological systems(D) integumentary system(E), and neurological (F)

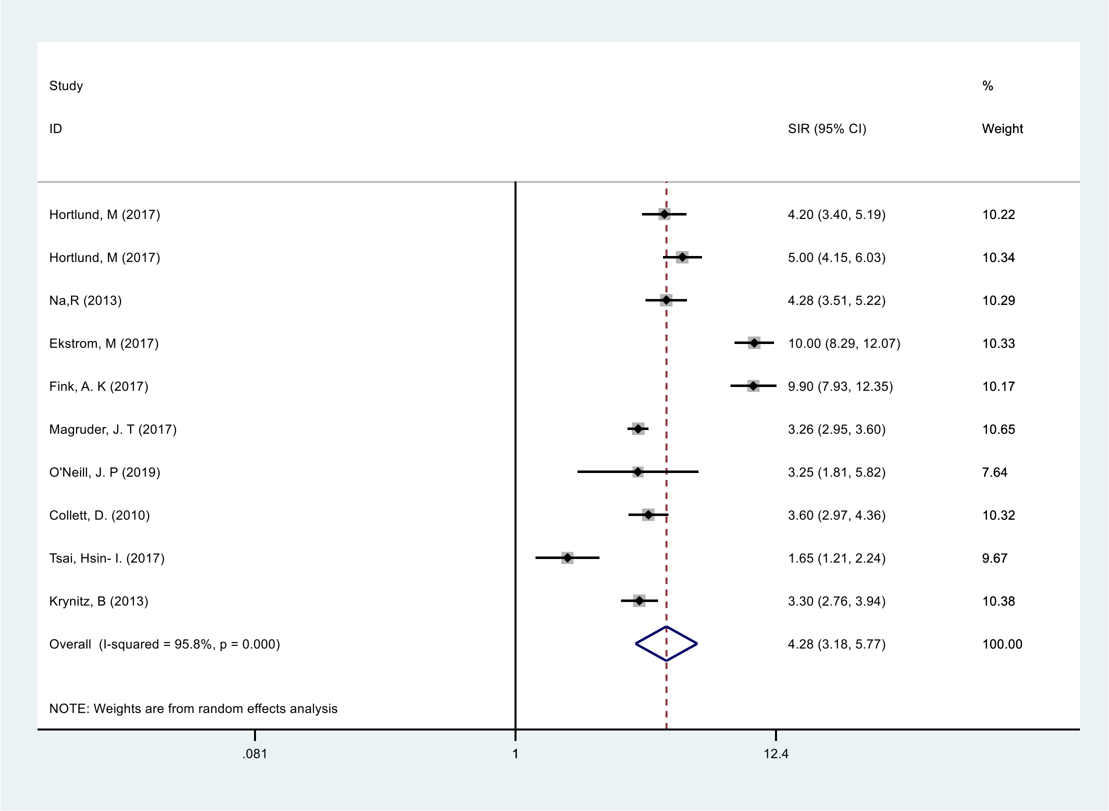

Figure S3: Forest plot for all cancers in lung transplantation.

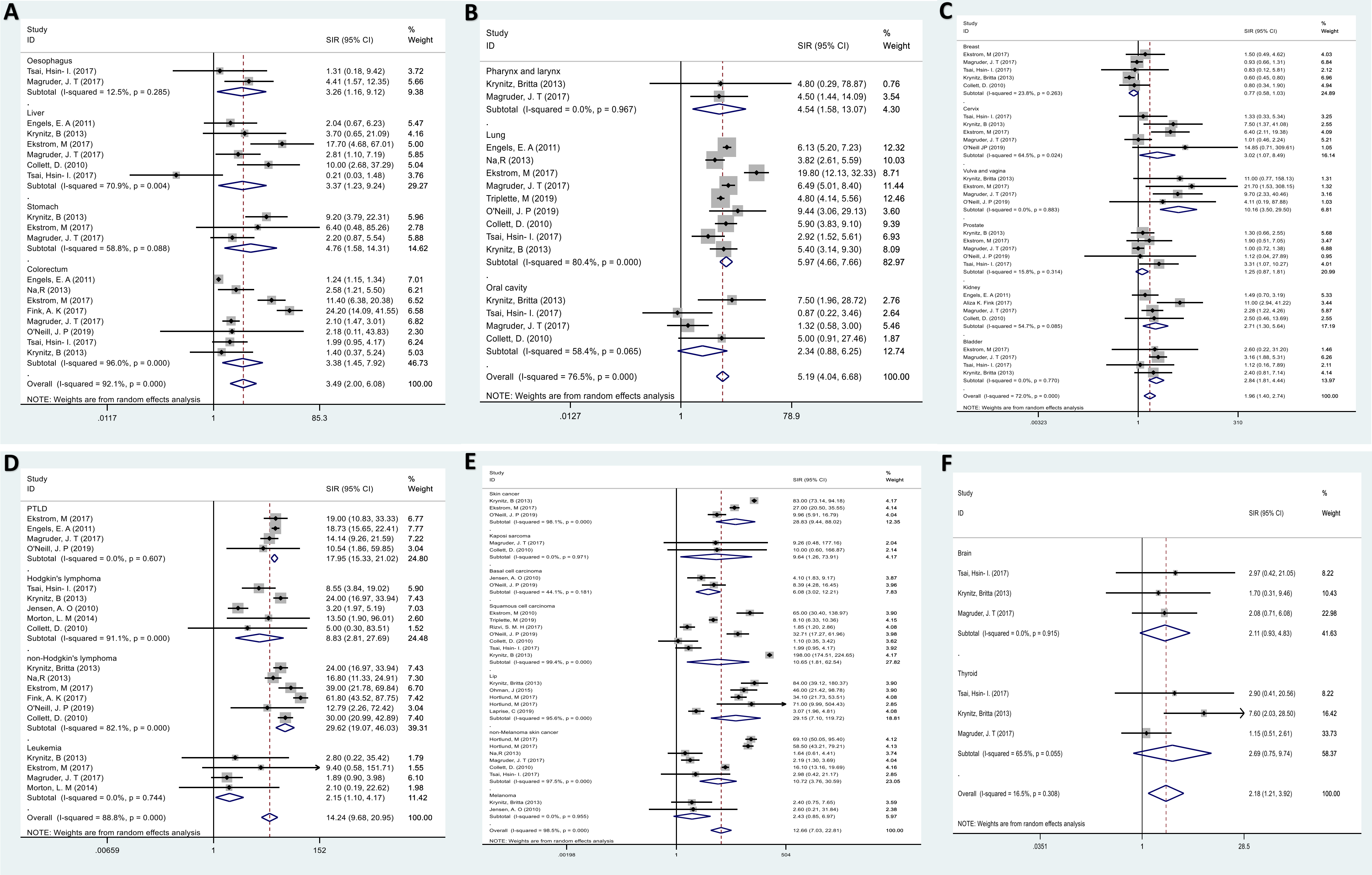

Figure S4: Forest plots for different systems cancers in lung transplantation. Digestive system (A), respiratory system (B), reproductive and urinary systems(C), lymphatic and hematological systems(D) integumentary system(E), and neurological (F).

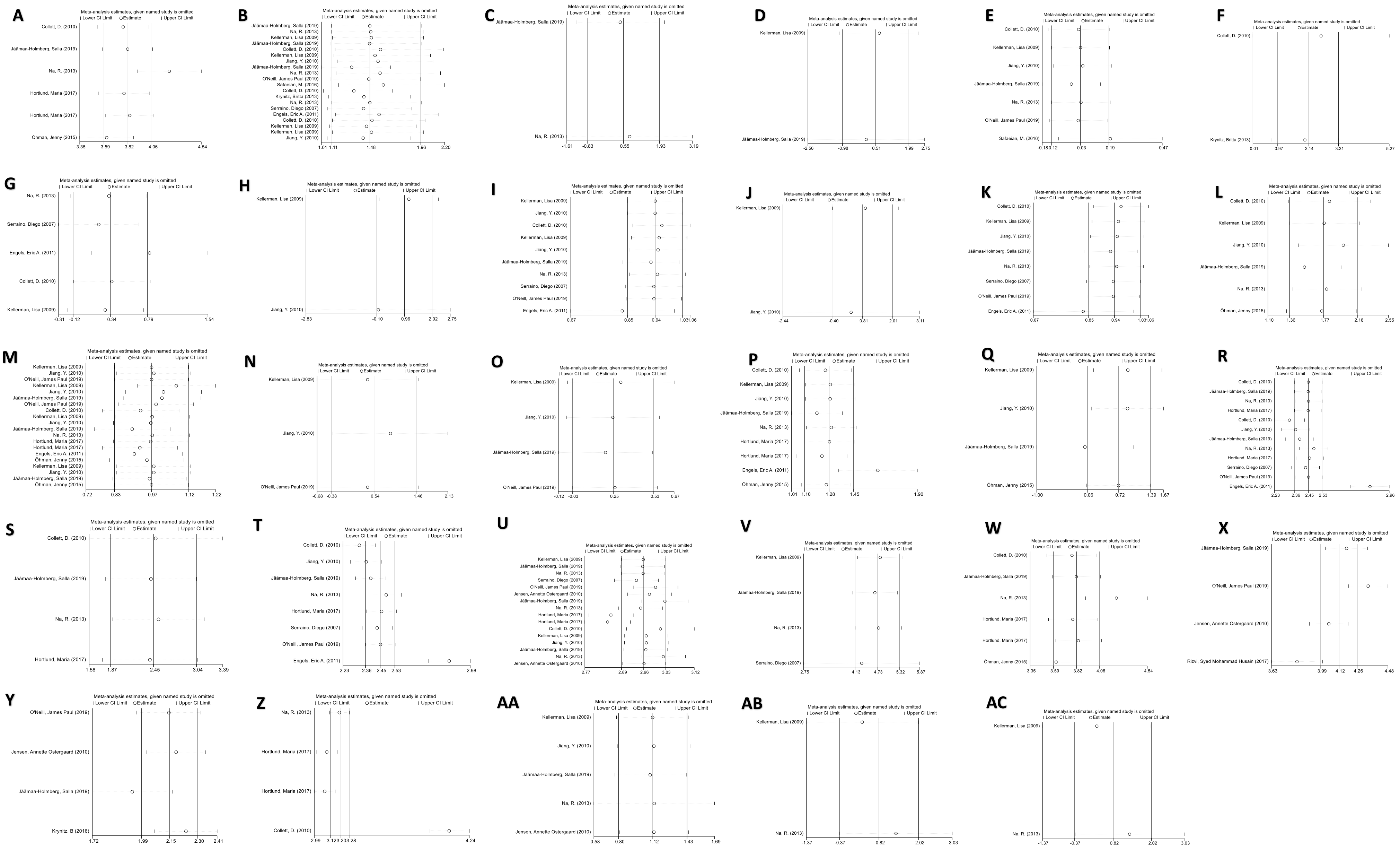

Figure S5: Sensitivity analysis for cancers in heart transplantation. All cancer (A), digestive system (B), oesophagus (C), stomach (D), colorectum (E), anus (F), liver (G), pancreas (H), respiratory system (I), pharynx and larynx (J), lung(K), oral cavity (L), reproductive and urinary systems (M), breast (N), prostate (O), kidney (P), bladder (Q), lymphatic and hematological systems (R), Hodgkin's lymphoma (S), non-Hodgkin's lymphoma (T), integumentary system (U), Kaposi sarcoma (V), lip (W), squamous cell carcinoma (X), basal cell carcinoma (Y), non-melanoma skin cancer (Z), melanoma (AA), neurological (AB), and brain (AC).

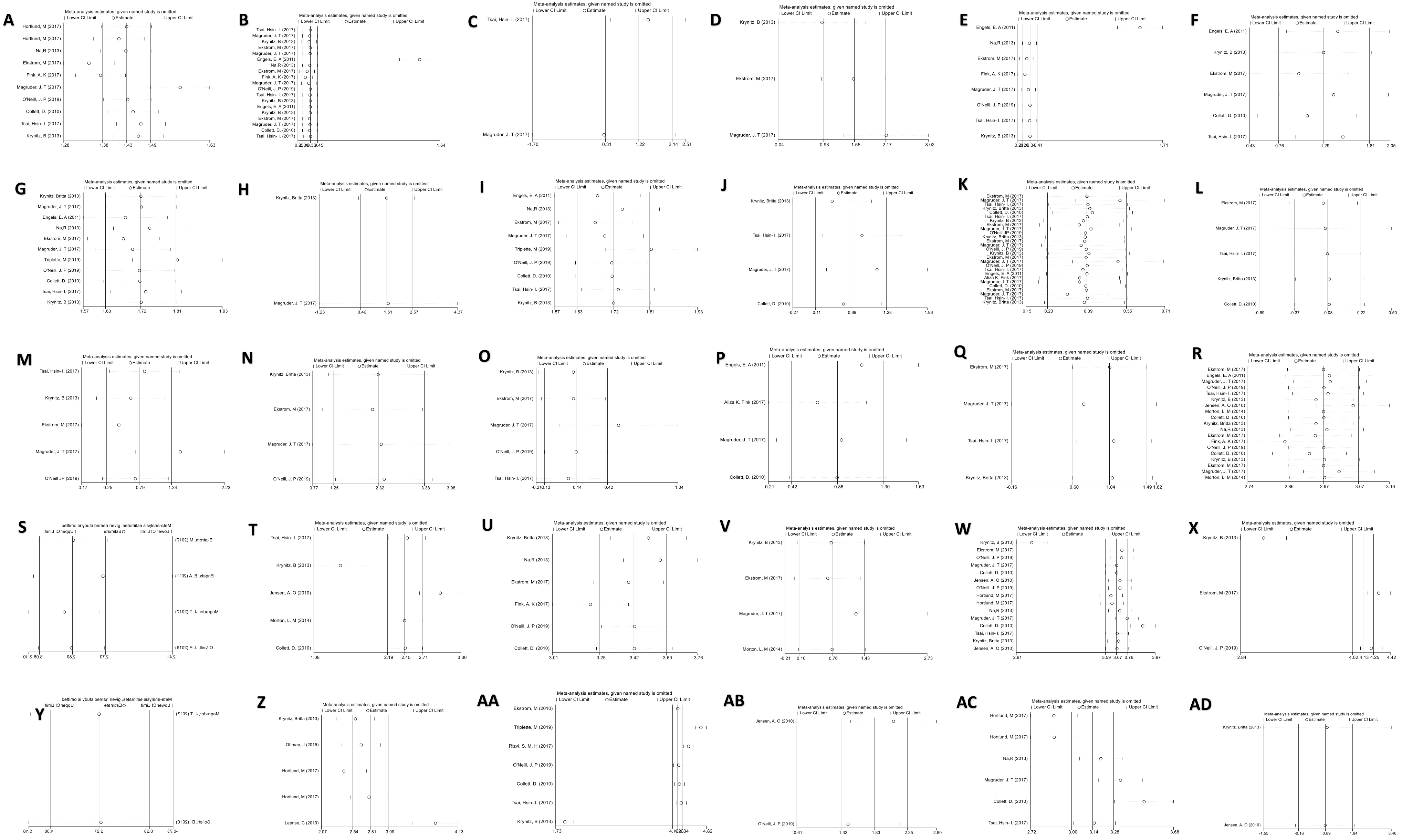
A
B
C
D
E
F
G
H
I
J
K
L
M
N
O
P
Q
R
S
T
U
V
W
X
Y
Z
AA
AB
AC
AD
Meta-analysis estimates, given named study is omitted
Lower CI Limit
Estimate
Upper CI Limit

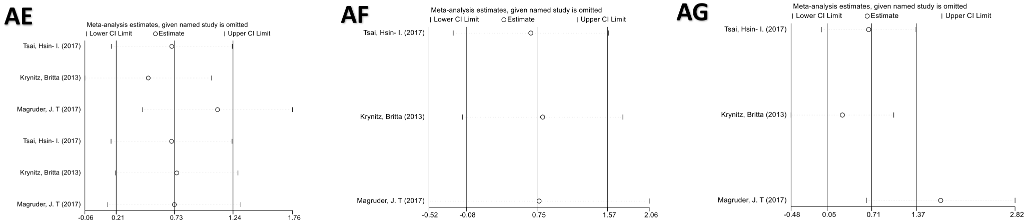

Figure S6: Sensitivity analysis for cancers in lung transplantation. All cancer (A), digestive system (B), oesophagus (C), stomach (D), colorectum (E), liver (F), respiratory system (G), pharynx and larynx (H), lung(I), oral cavity (J), reproductive and urinary systems (K), breast (L), cervix (M), vulva and vagina (N), prostate (O), kidney (P), bladder (Q), lymphatic and hematological systems (R), PTLD (S), Hodgkin's lymphoma (T), non-Hodgkin's lymphoma (U), leukemia (V), integumentary system (W), skin cancer (X), Kaposi sarcoma (Y), lip (Z), squamous cell carcinoma (AA), basal cell carcinoma (AB), non-melanoma skin cancer (AC), melanoma (AD), neurological (AE), brain (AF), and thyroid (AG).

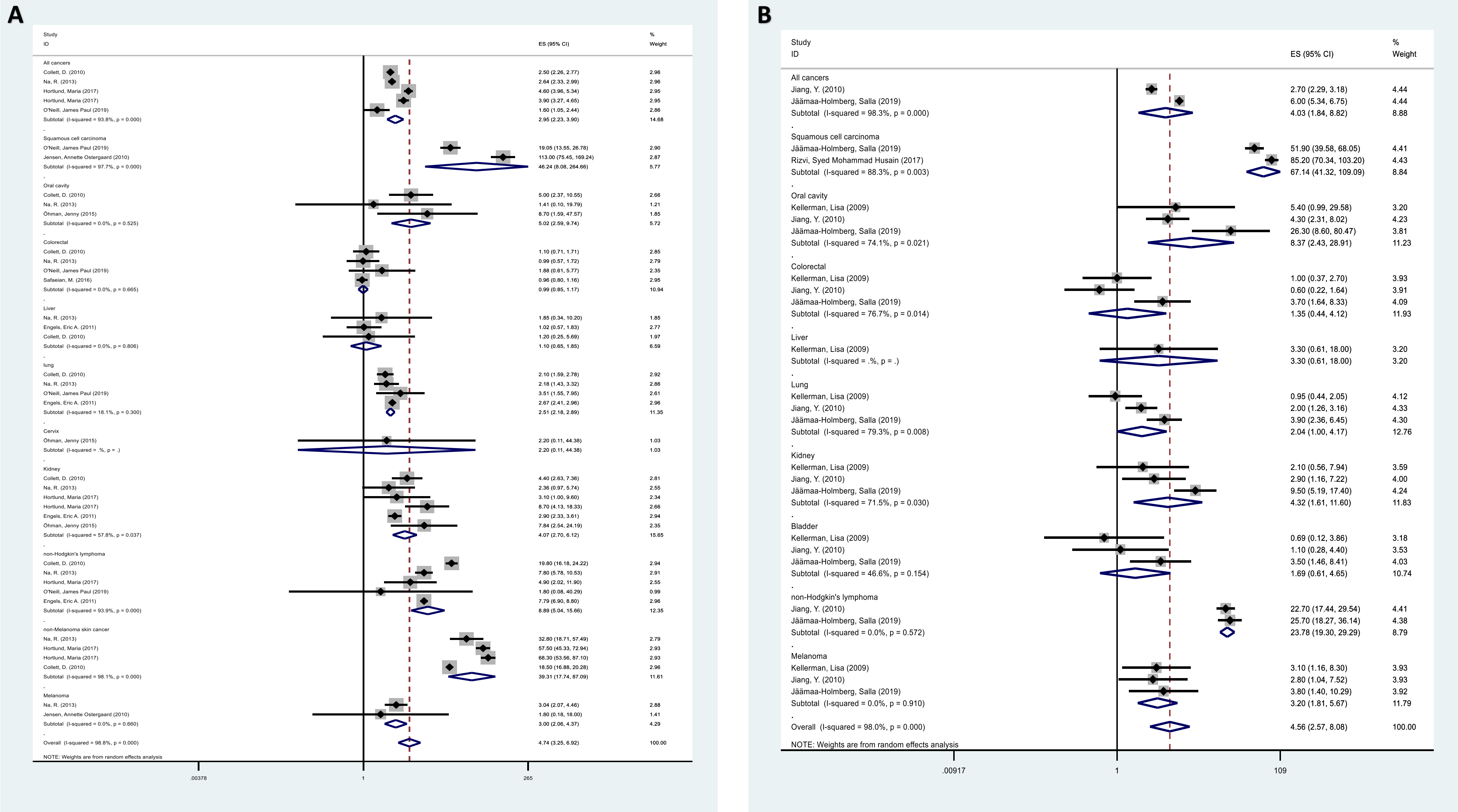

Figure S7: Forest plots for cancers in heart transplant recipients at different ages. Younger than 50 years (A), and older than 50 years (B).

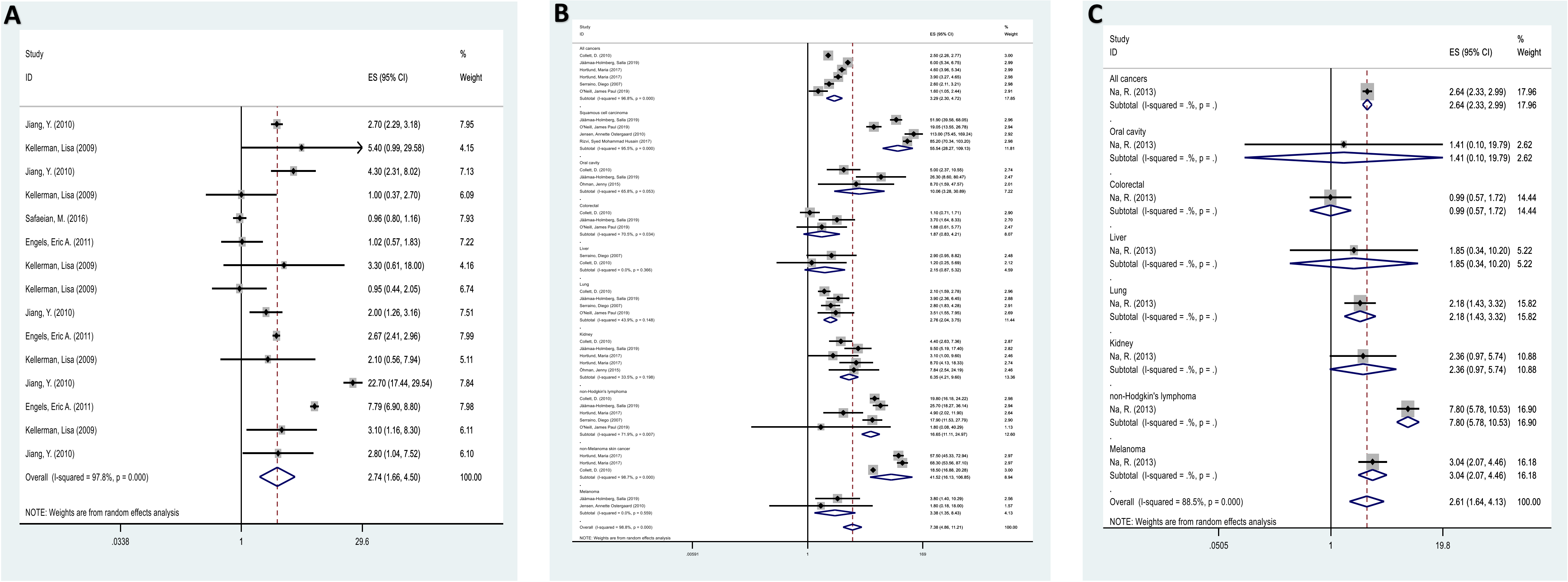

Figure S8: Forest plots for cancers in heart transplant recipients at different regions. South American (A), European (B), and Oceania (C).

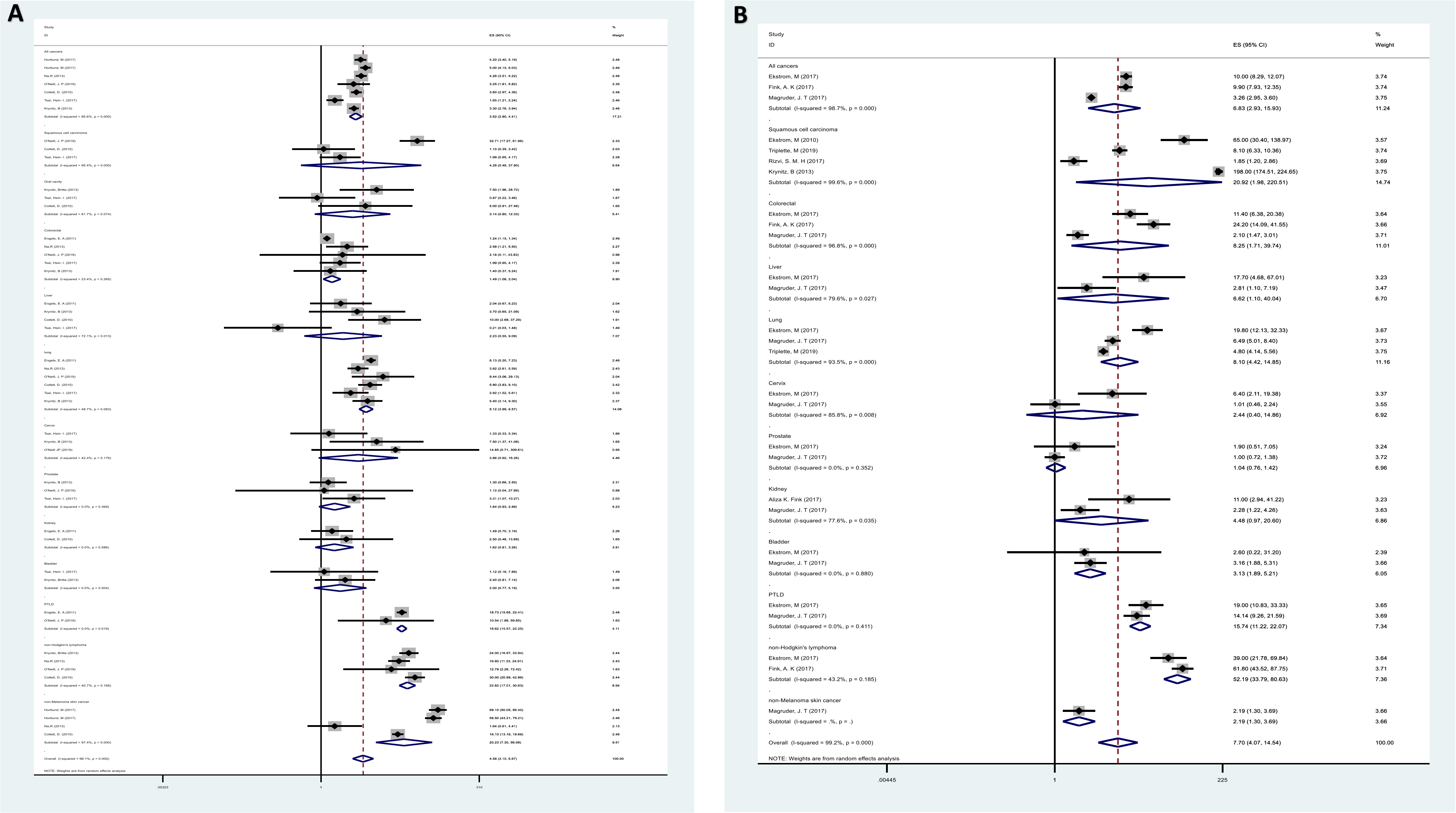

Figure S9: Forest plots for cancers in lung transplant recipients at different ages. Younger than 50 years (A), and older than 50 years (B).

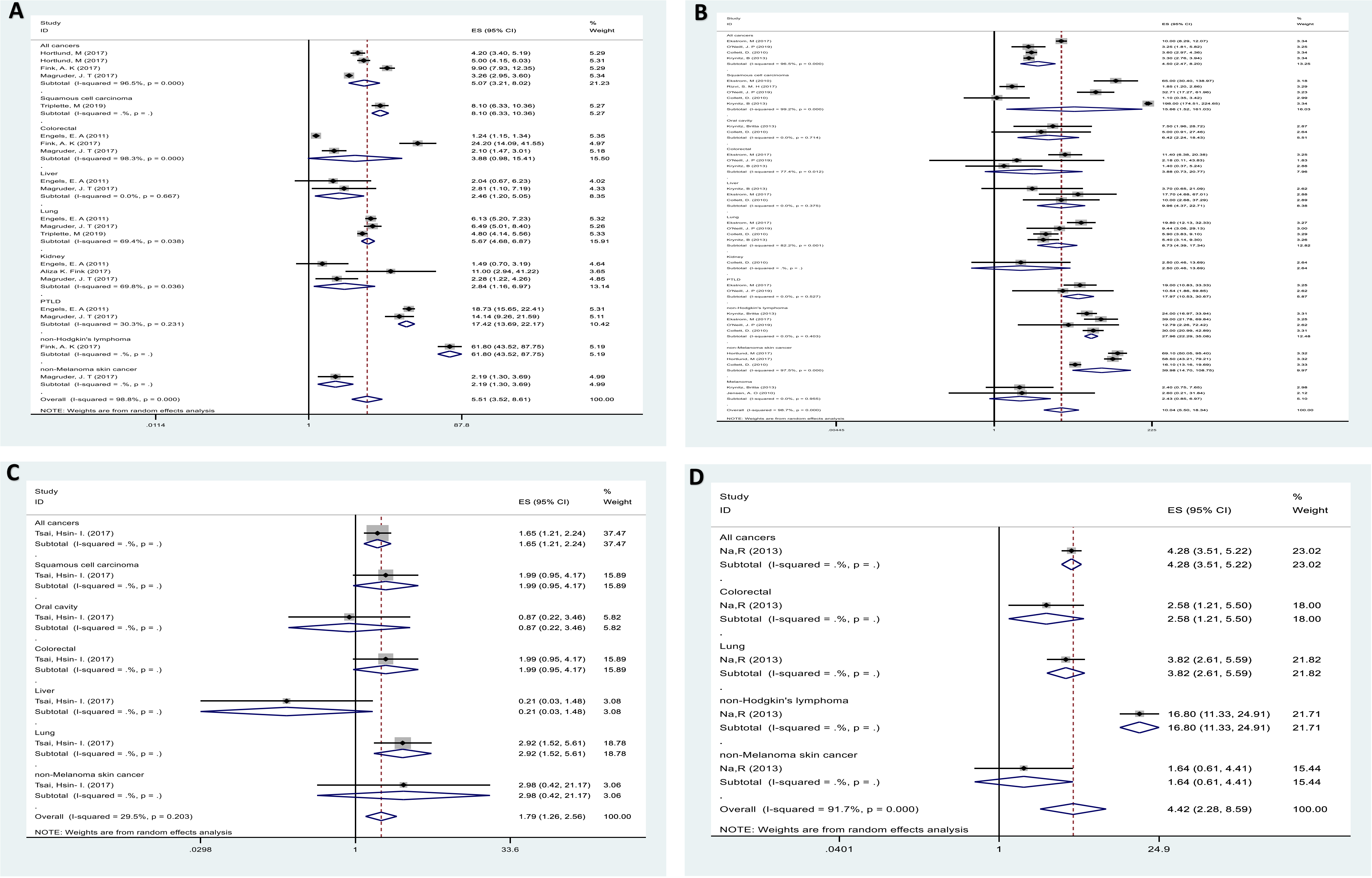

Figure S10: Forest plots for cancers in lung transplant recipients at different regions. South American (A), European (B), Asian (C) and Oceania (D).

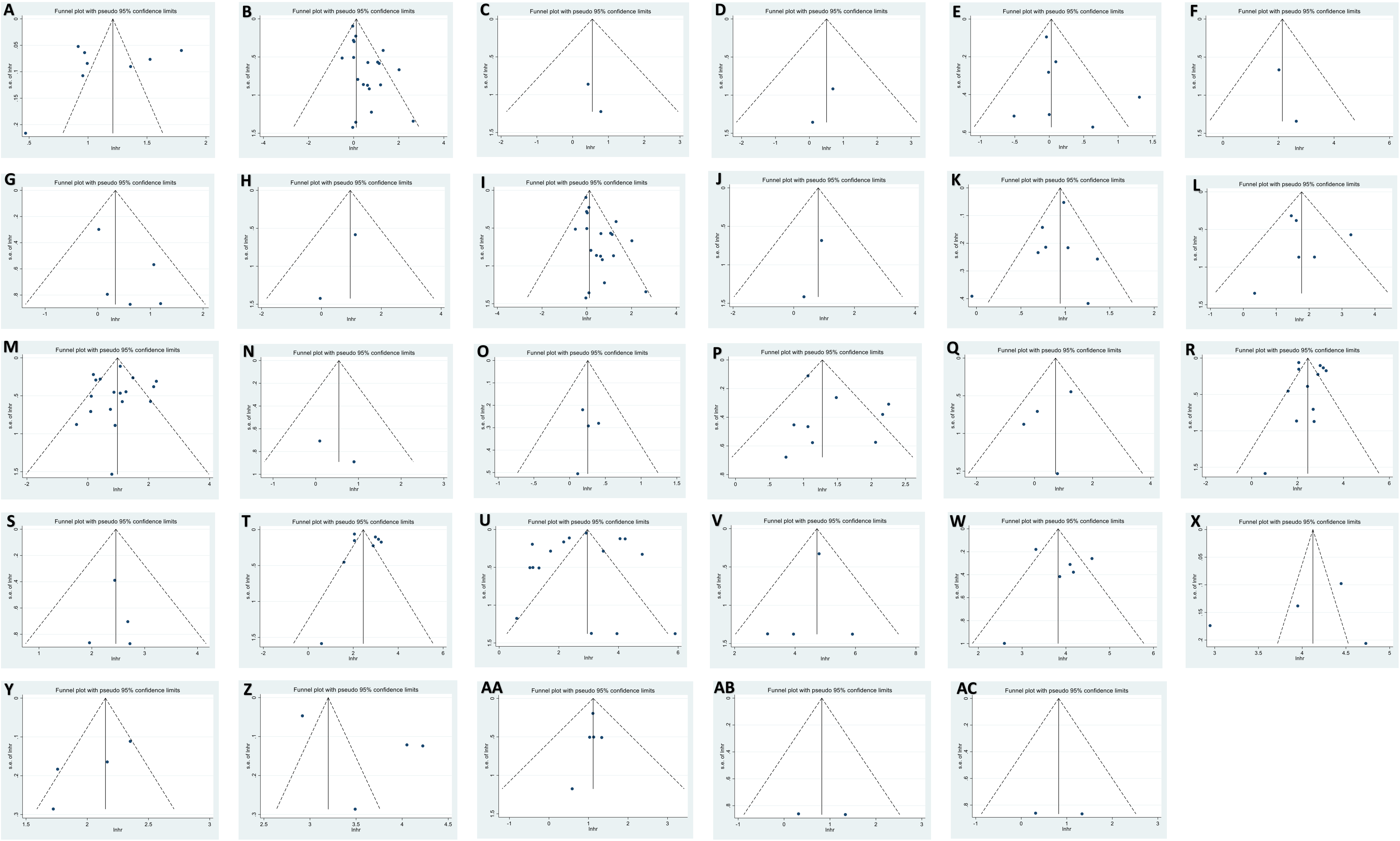

Figure S11: Sensitivity analysis for cancers in heart transplantation. All cancer (A), digestive system (B), oesophagus (C), stomach (D), colorectum (E), anus (F), liver (G), pancreas (H), respiratory system (I), pharynx and larynx (J), lung(K), oral cavity (L), reproductive and urinary systems (M), breast (N), prostate (O), kidney (P), bladder (Q), lymphatic and hematological systems (R), Hodgkin's lymphoma (S), non-Hodgkin's lymphoma (T), integumentary system (U), Kaposi sarcoma (V), lip (W), squamous cell carcinoma (X), basal cell carcinoma (Y), non-melanoma skin cancer (Z), melanoma (AA), neurological (AB), and brain (AC).

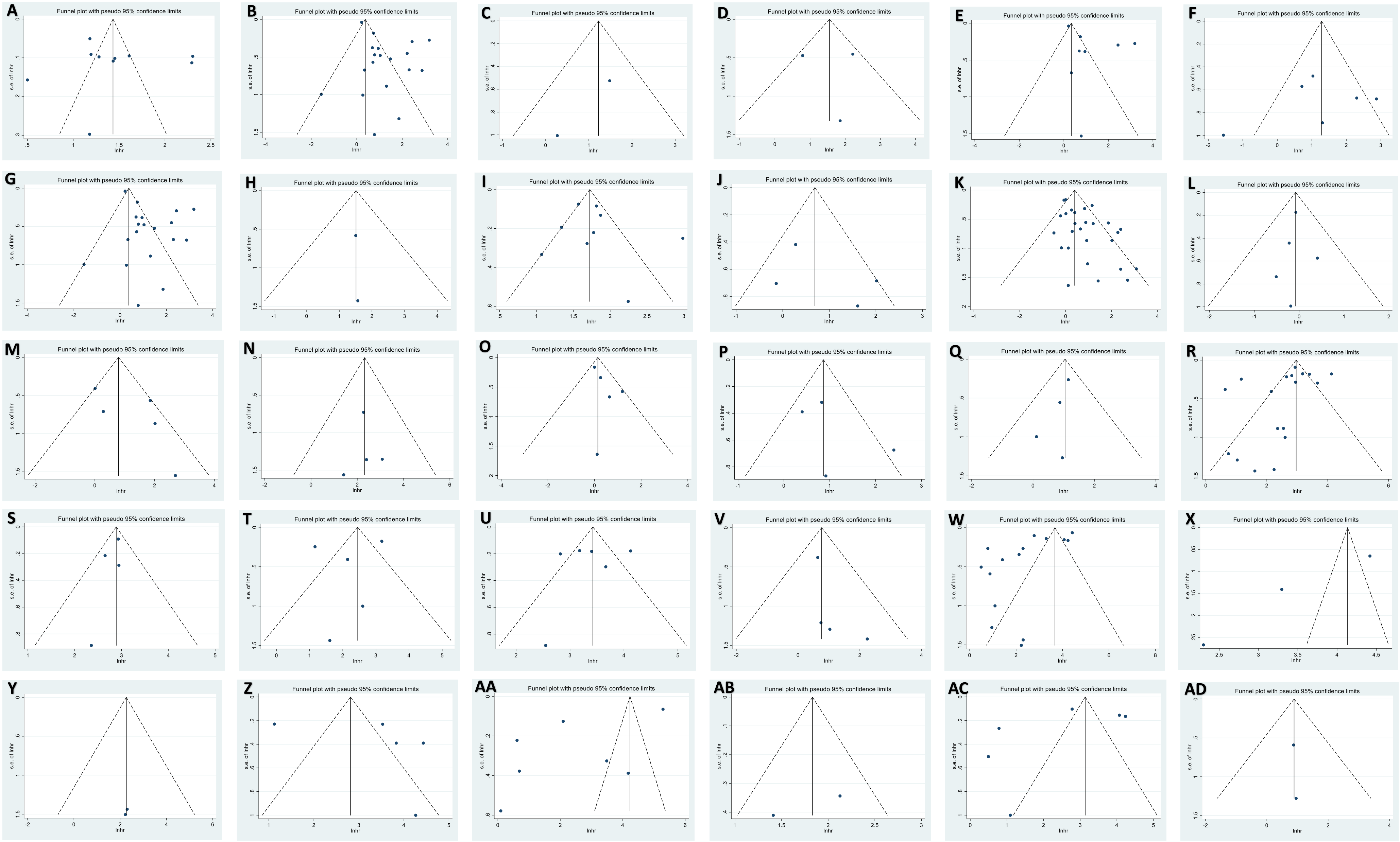
A
Funnel plot with pseudo 95% confidence limits
s.e. of lnhr
lnhr
B
Funnel plot with pseudo 95% confidence limits
s.e. of lnhr
lnhr
C
Funnel plot with pseudo 95% confidence limits
s.e. of lnhr
lnhr
D
Funnel plot with pseudo 95% confidence limits
s.e. of lnhr
lnhr
E
Funnel plot with pseudo 95% confidence limits
s.e. of lnhr
lnhr
F
Funnel plot with pseudo 95% confidence limits
s.e. of lnhr
lnhr
G
Funnel plot with pseudo 95% confidence limits
s.e. of lnhr
lnhr
H
Funnel plot with pseudo 95% confidence limits
s.e. of lnhr
lnhr
I
Funnel plot with pseudo 95% confidence limits
s.e. of lnhr
lnhr
J
Funnel plot with pseudo 95% confidence limits
s.e. of lnhr
lnhr
K
Funnel plot with pseudo 95% confidence limits
s.e. of lnhr
lnhr
L
Funnel plot with pseudo 95% confidence limits
s.e. of lnhr
lnhr
M
Funnel plot with pseudo 95% confidence limits
s.e. of lnhr
lnhr
N
Funnel plot with pseudo 95% confidence limits
s.e. of lnhr
lnhr
O
Funnel plot with pseudo 95% confidence limits
s.e. of lnhr
lnhr
P
Funnel plot with pseudo 95% confidence limits
s.e. of lnhr
lnhr
Q
Funnel plot with pseudo 95% confidence limits
s.e. of lnhr
lnhr
R
Funnel plot with pseudo 95% confidence limits
s.e. of lnhr
lnhr
S
Funnel plot with pseudo 95% confidence limits
s.e. of lnhr
lnhr
T
Funnel plot with pseudo 95% confidence limits
s.e. of lnhr
lnhr
U
Funnel plot with pseudo 95% confidence limits
s.e. of lnhr
lnhr
V
Funnel plot with pseudo 95% confidence limits
s.e. of lnhr
lnhr
W
Funnel plot with pseudo 95% confidence limits
s.e. of lnhr
lnhr
X
Funnel plot with pseudo 95% confidence limits
s.e. of lnhr
lnhr
Y
Funnel plot with pseudo 95% confidence limits
s.e. of lnhr
lnhr
Z
Funnel plot with pseudo 95% confidence limits
s.e. of lnhr
lnhr
AA
Funnel plot with pseudo 95% confidence limits
s.e. of lnhr
lnhr
AB
Funnel plot with pseudo 95% confidence limits
s.e. of lnhr
lnhr
AC
Funnel plot with pseudo 95% confidence limits
s.e. of lnhr
lnhr
AD
Funnel plot with pseudo 95% confidence limits
s.e. of lnhr
lnhr

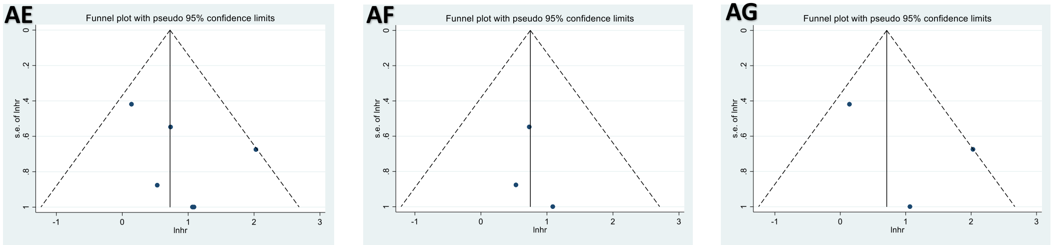

Figure S12: Sensitivity analysis for cancers in lung transplantation. All cancer (A), digestive system (B), oesophagus (C), stomach (D), colorectum (E), liver (F), respiratory system (G), pharynx and larynx (H), lung(I), oral cavity (J), reproductive and urinary systems (K), breast (L), cervix (M), vulva and vagina (N), prostate (O), kidney (P), bladder (Q), lymphatic and hematological systems (R), PTLD (S), Hodgkin's lymphoma (T), non-Hodgkin's lymphoma (U), leukemia (V), integumentary system (W), skin cancer (X), Kaposi sarcoma (Y), lip (Z), squamous cell carcinoma (AA), basal cell carcinoma (AB), non-melanoma skin cancer (AC), melanoma (AD), neurological (AE), brain (AF), and thyroid (AG).
